# MicroRNA-7 inhibits the stemness of prostate cancer stem-like cells and tumorigenesis by repressing KLF4/PI3K/Akt/p21 pathway

**DOI:** 10.18632/oncotarget.4447

**Published:** 2015-06-29

**Authors:** Yun-Li Chang, Pei-Jie Zhou, Lianzi Wei, Wang Li, Zhongzhong Ji, Yu-Xiang Fang, Wei-Qiang Gao

**Affiliations:** ^1^ State Key Laboratory of Oncogenes and Related Genes, Renji-Med X Clinical Stem Cell Research Center, Ren Ji Hospital, School of Medicine, Shanghai Jiao Tong University, Shanghai 200127, China; ^2^ School of Biomedical Engineering & Med-X Research Institute, Shanghai Jiao Tong University, Shanghai 200030, China; ^3^ Collaborative Innovation Center of Systems Biomedicine, Shanghai Jiao Tong University, Shanghai 200030, China

**Keywords:** miR-7, prostate cancer, cancer stem cells, tumorigenesis, KLF4, PI3K/Akt pathway

## Abstract

Up to now, the molecular mechanisms underlying the stemness of prostate cancer stem cells (PCSCs) are still poorly understood. In this study, we demonstrated that microRNA-7 (miR-7) appears to be a novel tumor-suppressor miRNA, which abrogates the stemness of PCSCs and inhibits prostate tumorigenesis by suppressing a key stemness factor KLF4. MicroRNA-7 is down-regulated in prostate cancer cells compared to non-tumorigenic prostate epithelial cells. Restoration of miR-7 suppresses the expression of the stemness factor KLF4 in PCSCs and inhibits prostate tumorigenesis both *in vitro* and *in vivo*. Interestingly, the suppression of the stemness of PCSCs by miR-7 is sustained for generations in xenografts. Analysis of clinical samples also revealed a negative correlation between miR-7 expression and prostate tumor progression. Mechanistically, overexpression of miR-7 may lead to a cell cycle arrest but not apoptosis, which seems achieved via suppressing the KLF4/PI3K/Akt/p21 pathway. This study identifies miR-7 as a suppressor of PCSCs' stemness and implicates its potential application for PCa therapy.

## INTRODUCTION

In recent years, cancer stem cell (CSC) hypothesis has provided novel insights into understanding of tumorigenic mechanisms [1]. CSCs have been identified in several types of cancers [2, 3] including prostate cancer (PCa) [4]. Thus, it is generally believed that more effective treatments for PCa patients can be achieved via targeting prostate cancer stem cells (PCSCs).

It has been documented that microRNAs (miRNAs) are involved in promoting or inhibiting the stemness of CSCs [5]. Aberrantly expressed miRNAs can cause dysregulation of specific signaling pathways that are associated with the functions of CSCs [6]. Therefore, identification of specific miRNAs that regulate PCSC properties would be helpful for expanding our understanding of molecular pathogenesis of PCa and designing better strategies for PCa treatment. Toward that direction, we paid particular attention to miR-7, which was initially discovered in *Drosophila* [7], due to its essential role in tumorigenesis [8]. Our previous work demonstrated that miR-7 inhibits liver cancer proliferation and metastasis by repressing the PI3K/Akt pathway [9]. In addition, miR-7 is reported to inhibit breast CSCs' stemness [10] by suppressing the STAT3 pathway. However, whether miR-7 is involved in prostate tumorigenesis and/or regulating PCSCs' stemness has not been determined.

In this study, we set forth to determine the potential role of miR-7 during prostate tumorigenesis. We found that restoration of miR-7 effectively inhibited PCSCs' stemness. Furthermore, this function on stemness inhibition could be sustained in xenograft experiments for generations. Importantly, we showed evidences that miR-7 inhibited PCSCs' stemness and prostate tumorigenesis by directly suppressing a key stemness factor KLF4 [11] and in turn inhibiting its downstream PI3K/Akt/p21 axis.

## RESULTS

### MiR-7 is down-regulated in PCa cells

In order to evaluate the role of miR-7 in PCa, we first investigated the relative miR-7 expression in human PCa cell lines vs non-tumorigeneic human prostatic epithelial cell lines. As shown in Supplementary Figure 1A, miR-7 expression was significantly reduced in all PCa cell lines, especially in PC3 (0.28 ± 0.05), implicating its potential tumor suppressive function in PCa.

### MiR-7 is down-regulated in CD44+CD133+ stem-like cells in PCa

As CD44+CD133+ subpopulation appears to possess CSC features in various types of cancers [12–15], we evaluated whether the CD44+CD133+ subpopulation displays CSC features in PCa and determined the expression levels of miR-7 in CD44+CD133+ vs CD44-CD133- cells. We isolated CD44+CD133+ and CD44-CD133- subpopulations from PC3-derived xenografts (Supplementary Figure 1B) and determined the expression levels of stemness factors in both subpopulations (Supplementary Figure 1C and 1D). We found that the expression levels of all the four stemness factors were significantly higher in CD44+CD133+ than CD44-CD133- subpopulations, suggesting that CD44+CD133+ cells possessed PCSC characteristics [16]. To validate this hypothesis, we carried out limited dilution analysis [17] to verify the CSC potential of CD44+CD133+ cells *in vivo*. While 100 CD44+CD133+ cells were sufficient for tumorigenesis, 10^4^ CD44-CD133- cells were required under the same conditions, suggesting that CD44+CD133+ cells had a significantly stronger ability for tumor initiation (Supplementary Figure 1E) and represented a stem-like cell feature. We then compared the expression level of miR-7 between CD44+CD133+ and CD44-CD133- cells (Supplementary Figure 1F). Notably, miR-7 was significantly reduced in CD44+CD133+ stem-like cells (0.22 ± 0.01). These findings suggested that miR-7 is closely correlated with the stemness of PCSCs.

### Restoration of miR-7 suppresses PCSCs' stemness

**Figure 1:**
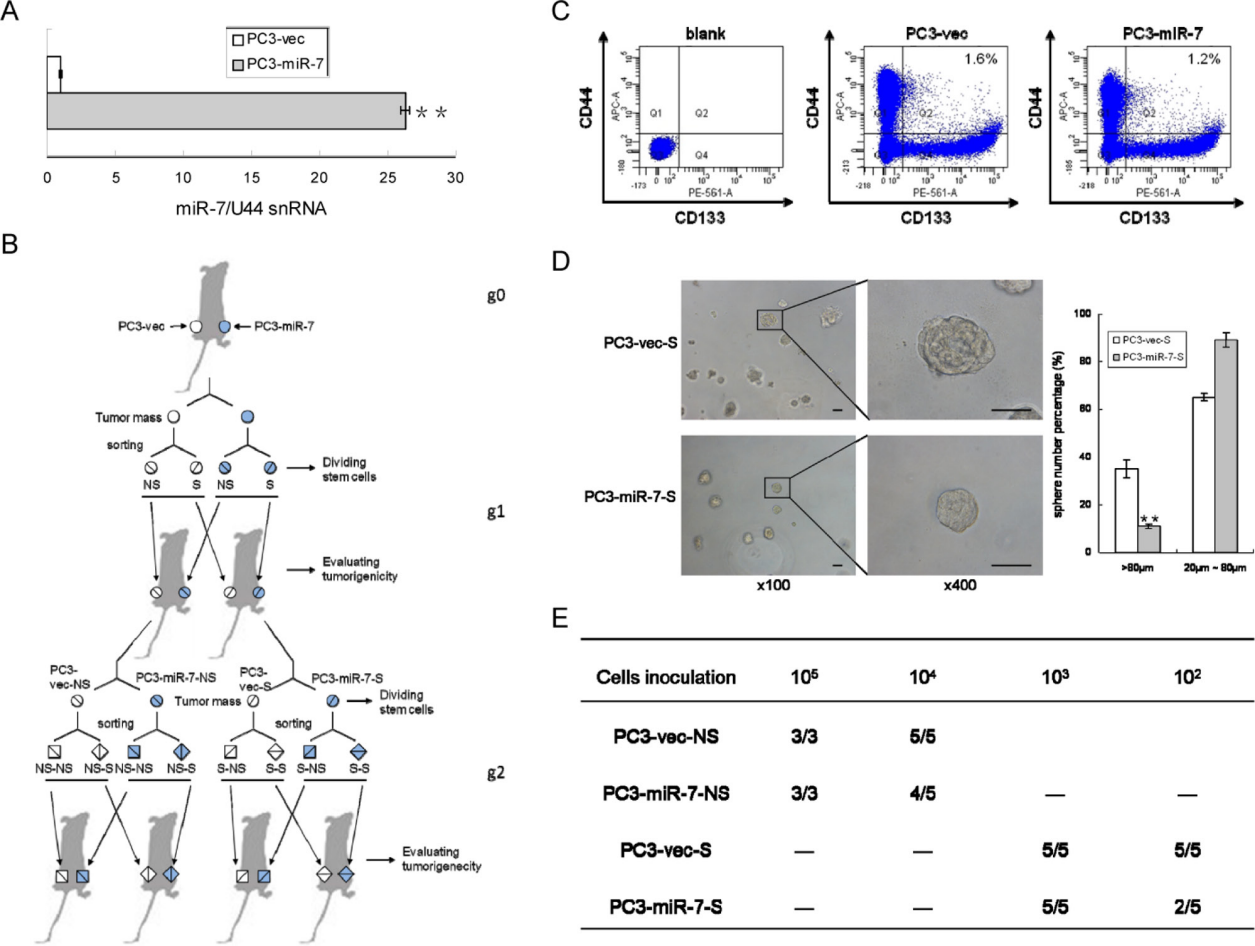
MiR-7 impairs the stemness of PCSCs **A.** Construction of PC3-miR-7 subclone cell line significantly increased miR-7 expression. **B.** Strategy for evaluating the function of miR-7 on impairing the stemness of PCSCs by limited dilution assay. NS: non stem-like cells, S: stem-like cells. **C.** Restoration of miR-7 decreases the proportion of PCSCs compared to the control. PCSCs are sorted from PC3-miR-7 and PC3-vec derived grafts respectively. Blank: without antibody incubation. **D.** Restoration of miR-7 inhibits sphere formation in PCSCs *in vitro*. Magnification: × 100; × 400, Bar: 50 μm. **E.** Restoration of miR-7 suppresses the *in vivo* tumorigenesis of both stem-like and non stem-like cells in PCa (numbers of tumor formed vs numbers of mice inoculated in a group, —: no experiment carried out). Data are represented as mean ± SEM. **:*p* < 0.01

In order to explore the regulatory mechanisms of miR-7 in PCSCs, we established PC3-miR-7 and PC3-vec (control) subclone cell line and determined a 27-fold overexpression of miR-7 in PC3-miR-7 vs PC3-vec cells (Figure 1A). To validate the inhibitory effect of miR-7 on the PCSCs' stemness, we carried out limited dilution assay (Figure 1B) after the CD44+CD133+ (stem-like cells) and CD44-CD133- cells (non stem-like cells) were sorted from PC3-vec or PC3-miR-7 derived xenografts (parental generation grafts, g0 grafts, Figure 1C) and passaged them subcutaneously for two generations (1st generation, g1 and 2nd generation, g2). As shown in Figure 1C, the proportion of stem-like cells was decreased in PC3-miR-7 g0 graft, which indicated an impairment to maintain the CSC pool after restoration of miR-7.

We further evaluated the tumorigenic capability of stem-like and non-stem-like cells (named as PC3-miR-7-NS and PC3-vec-NS cell respectively) *in vitro* and *in vivo*, which were sorted from PC3-miR-7 or PC3-vec derived g0 grafts. We found that the proportion of large spheres (diameter > 80 μm) formed from PC3-miR-7-S cells (11.2% ± 1.12%) was significantly lower than those from PC3-vec-S cells (35.3% ± 3.82%) *in vitro* (Figure 1D). By limited dilution analysis, both PC3-miR-7-NS and PC3-miR-7-S cells had a poorer ability to initiate tumorigenesis and formed smaller xenografts than PC3-vec-NS and PC3-vec-S cells, respectively (Figure 1E, Supplementary Figure 2A and 2B). These results demonstrated that restoration of miR-7 expression in PC3 suppressed the PCSCs' stemness and in turn impaired tumorigenesis in next generation.

### The inhibition of miR-7 on PCSCs' stemness continues for generations in xenografts

**Figure 2:**
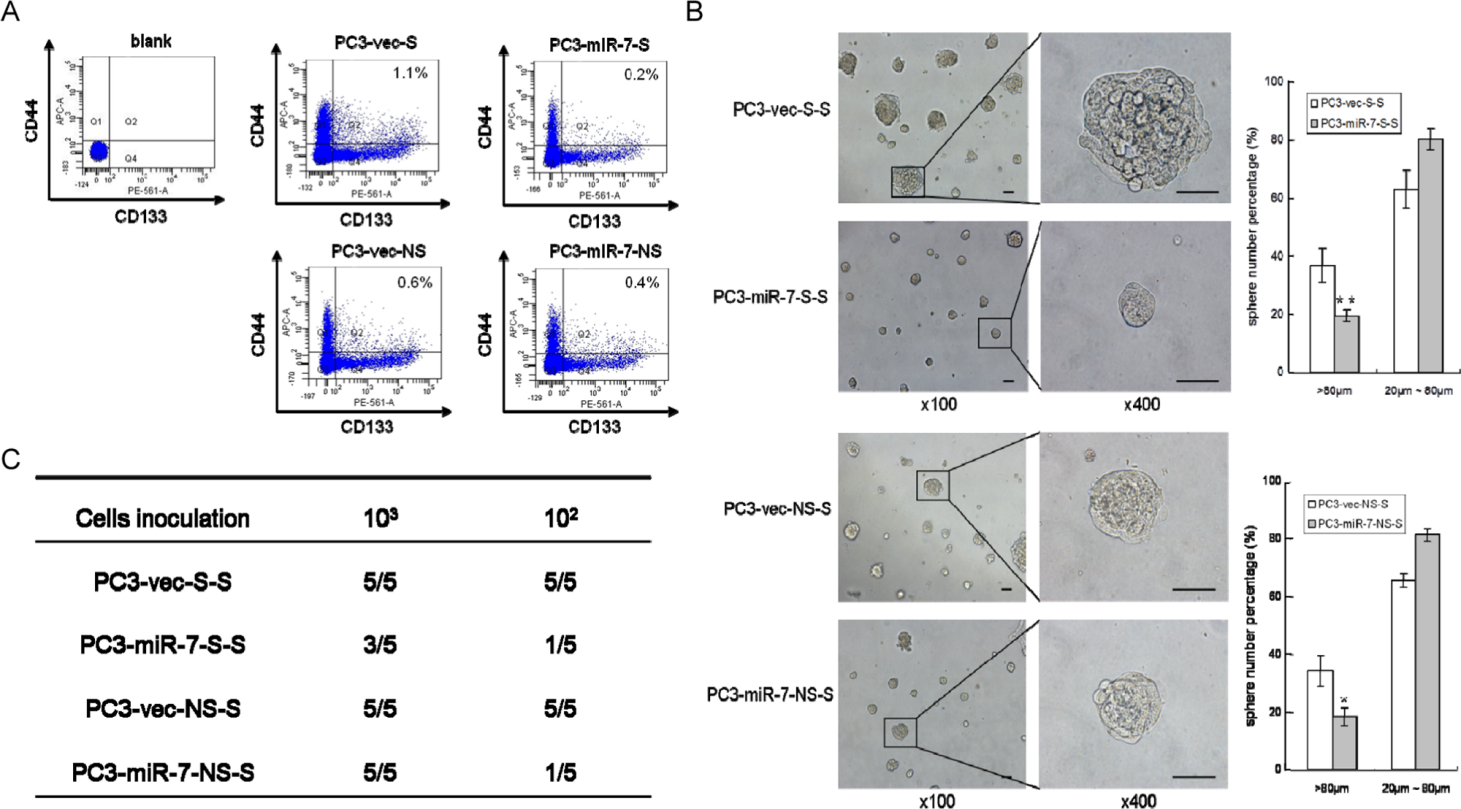
Restoration of miR-7 continuously inhibits the stemness of PCSCs for generations **A.** Stem-like cells are sorted from PC3-vec-S, PC3-miR-7-S, PC3-vec-NS and PC3-miR-7-NS derived grafts respectively. The proportion of 2^nd^ generation (g2) stem-like cells is further decreased by miR-7 restoration. Blank: without antibody incubation. **B.** Restoration of miR-7 inhibits sphere formation of g2 stem-like cells *in vitro*. Magnification: × 100; × 400, Bar: 50 μm. **C.** Restoration of miR-7 suppresses the *in vivo* tumorigenesis of g2 stem-like cells derived grafts (numbers of tumor formed vs numbers of mice inoculated in a group). Data are represented as mean ± SEM. *:*p* < 0.05; **:*p* < 0.01

We further investigated whether the impairment of PCSCs' stemness by miR-7 restoration could be sustained by generations. Stem-like cells were sorted again from either PC3-miR-7-S or PC3-vec-S derived g1 grafts, which were named PC3-miR-7-S-S and PC3-vec-S-S cells (2^nd^ generation, g2) respectively (Figure 1B). We found that the proportion of PC3-miR-7-S-S cells was further reduced than the control cells (0.2% vs 1.1%, *p* < 0.01), which indicated a continuous inhibition of stem cell pool charges by miR-7 restoration (Figure 2A). We further found that the proportion of PC3-miR-7-S-S derived large spheres was significantly decreased (19.6% ± 2.03% vs 36.7% ± 5.82%, *p* < 0.01), which indicated a continuous inhibition of sphere formation *in vitro* (Figure 2B). Meanwhile PC3-miR-7-S-S cells showed a lower tumor-forming rate and slower proliferation than PC3-vec-S-S cells *in vivo* (Figure 2C, Supplementary Figure 2C and 2D). These results indicated that restoration of miR-7 had a sustained effect on inhibition of PCSCs' stemness and impaired tumorigenesis for generations.

On the other hand, stem-like cells could also be sorted from PC3-miR-7-NS and PC3-vec-NS cells derived grafts (Figure 2A), named PC3-miR-7-NS-S and PC3-vec-NS-S cells respectively. We found that miR-7 maintained its capability to impair the sphere formation *in vitro* (Figure 2B) and tumorigenesis *in vivo* (Figure 2C, Supplementary Figure 2C and 2D) of these g2 grafts. These observations indicated that the impairment of stemness was persistent by miR-7 restoration no matter whether PCSCs in the next generation were derived from stem-like or non stem-like cells in the previous generation.

### Krüppel-like factor 4 (KLF4) is a functional target of miR-7 in PCa

**Figure 3:**
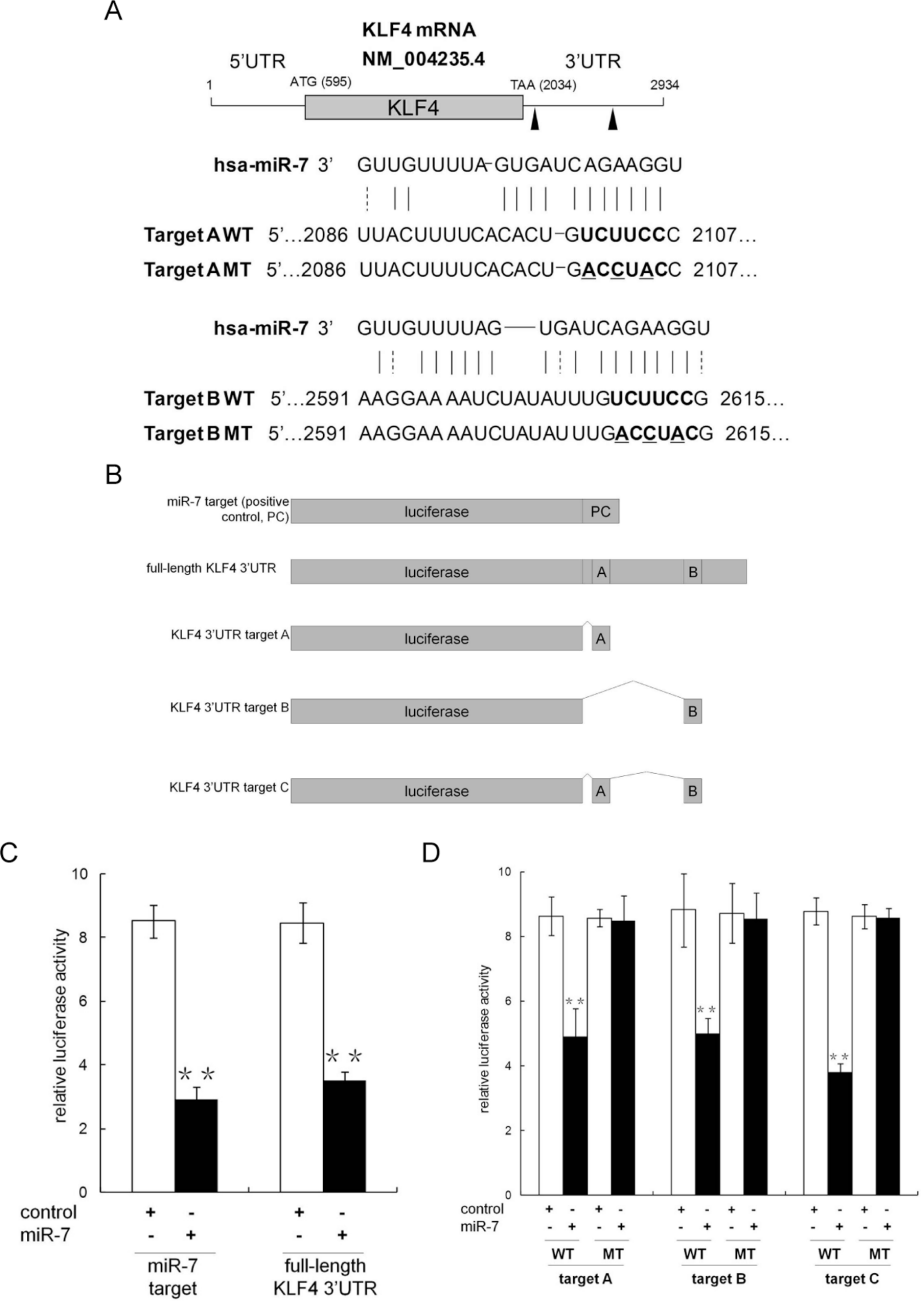
Identification of KLF4 as a target of miR-7 in PCa **A.** There are two miR-7 binding sites harbored in KLF4 mRNA 3′UTR by bioinformatic assay. **B.** Construction of relevant luciferase reporter vectors. **C.** Presence of full length KLF4 mRNA 3′UTR significantly suppresses the relative luciferase activity, which indicates an interaction between miR-7 and the 3′UTR. **D.** Both two binding sites effectively suppress the relative luciferase activity, which indicates that miR-7 inhibits KLF4 expression at post-transcriptional level via these binding sites. Data are represented as mean ± SEM. **:*p* < 0.01

Given the observation that miR-7 was suppressed in PCSCs and restoration of miR-7 impaired PCSCs' stemness for generations, we asked whether any key stemness factors are inhibited by miR-7. Using TargetScan (http://www.targetscan.org) and microRNA database (http://www.microrna.org), we identified Krüppel-like factor 4 (KLF4), but neither OCT4 nor Sox2 and Nanog, as a likely target of miR-7 because it contains two putative miR-7 binding sites in its 3′UTR (Figure 3A). To explore the potential suppression of KLF4 by miR-7, we generated a series of luciferase reporter vectors (Figure 3B). Our results indicated that miR-7 significantly reduced the relative luciferase activity when co-transfected with reporter vectors harboring full-length KLF4 3′UTR or the positive control (Figure 3C). We further evaluated the contribution of each putative miR-7 target sites and found that relative luciferase activity was reduced to 56.7% ± 10.3% or 56.0% ± 5.2% when the reporter vectors harboring putative mir-7 binding site A or B (but not the corresponding mutants) were co-transfected with miR-7 (Figure 3D). When two putative binding sites were integrated into an artificial binding site C, we found that relative luciferase activity was reduced to 43.4% ± 3.2% in the presence of miR-7, which was similar to what we observed with KLF4 3′UTR (Figure 3D). Collectively, these findings indicate that KLF4 is a specific target of miR-7 and that both of the two binding sites are functional sites for the interaction with miR-7 in PCa.

### KLF4 was continuously suppressed by miR-7 restoration for generations

To further validate the inhibition of miR-7 on KLF4, KLF4 expression in PC3-miR-7 and PC3-vec cells were assessed (Supplementary Figure 3A). KLF4 expression was significantly down-regulated in PC3-miR-7 cells, indicating that miR-7 inhibited KLF4 expression in PCa. We then evaluated KLF4 expression in PC3-miR-7-S compared to PC3-vec-S cells. Our data showed that KLF4 expression was significantly decreased in PC3-miR-7-S vs PC3-vec-S cells (Supplementary Figure 3B), indicating that suppression of PCSCs' stemness by miR-7 restoration was accomplished by inhibition of KLF4 expression. We further assessed the KLF4 expression in PC3-miR-7-S-S vs PC3-vec-S-S cells and PC3-miR-7-NS-S vs PC3-vec-NS-S cells respectively. We found that miR-7 continuously suppressed KLF4 expression in PCSCs in the next generation no matter whether they were derived from stem-like or non stem-like cells in the previous generation (Supplementary Figure 3C and 3D). These findings demonstrated that continuous suppression on the PCSCs' stemness by miR-7 restoration is achieved through sustained inhibition on KLF4 expression.

### Overexpression of KLF4 coding sequence rescues the suppression of PCSCs' stemness by miR-7 restoration

In order to further explore the inhibition of miR-7 on PCSCs' stemness by directly suppressing KLF4, we employed a lentivirus vector to overexpress KLF4 coding sequence (without 3′UTR) into PC3-miR-7 cells so that its expression would not be interfered by miR-7. By this rescue assay we wondered whether above phenotypes we observed could be reversed by KLF4 rescue. We also wanted to further investigate whether miR-7 impaired PCSCs' stemness by directly inhibiting its target KLF4 (Supplementary Figure 4). First we found that KLF4 expression was significantly recovered after infection of lentivirus vector containing KLF4 coding sequence (without 3′UTR, Supplementary Figure 4A) other than control. We here named relevant cells PC3-miR-7-KLF4 and PC3-miR-7-con (as control cells) respectively. Second, after stem-like cells were sorted from PC3-miR-7-con or PC3-miR-7-KLF4 derived xenografts (named PC3-miR-7-con-S and PC3-miR-7-KLF4-S respectively, Supplementary Figure 4B), we found that the proportion of stem-like cells was improved over 2-folds after KLF4 rescue (Supplementary Figure 4B). KLF4 expression was also increased in PC3-miR-7-KLF4-S cells (Supplementary Figure 4C). Third we found that the proportion of large spheres (diameter > 80 μm) formed from PC3-miR-7-KLF4-S cells (38.2% ± 2.52%) was significantly higher than those from PC3-miR-7-con-S cells (17.6% ± 5.62%) *in vitro* (Supplementary Figure 4D). All these results indicated that overexpression of KLF4 coding sequence efficiently rescued the inhibition of PCSCs' stemness caused by miR-7 restoration.

### Impairment of stemness in PCSCs by miR-7 restoration is achieved by down regulating KLF4

To further determine whether the abrogation of PCSCs' stemness by miR-7 restoration is achieved via inhibition of its target KLF4, we established PC3-shKLF4 vs PC3-con subclone cell lines to investigate whether phenotypes observed after KLF4 knock-down was similar to that after miR-7 restoration (Supplementary Figure 5A). By repeating the above experiments, we found that the proportion of stem-like cells (PC3-shKLF4-S and PC3-con-S cells, respectively) was also reduced in PC3-shKLF4 than PC3-con derived g0 grafts, which indicated that KLF4 knock-down had a direct correlation with the maintenance of the stem cell pool (Supplementary Figure 5B). Meanwhile PC3-shKLF4-S cells displayed a reduced capability to form large spheres *in vitro* (Supplementary Figure 5C). *In vivo* limited dilution assay also revealed that non stem-like cells (named PC3-shKLF4-NS and PC3-con-NS cells respectively) displayed a similar tumorigenesis capability, whereas the g1 grafts from PC3-shKLF4-NS cells remained smaller than the control (Supplementary Figure 5D). Importantly, PC3-shKLF4-S cells completely failed to form the next generation grafts even 10^3^ cells were inoculated (Supplementary Figure 5E), demonstrating that KLF4 knock-down directly impaired the maintenance of PCSCs' stemness. These findings indicated that restoration of miR-7 impaired the PCSCs' stemness by directly suppressing KLF4 expression and this function could be sustained for generations.

### Restoration of miR-7 inhibits overall prostatic tumor growth by specific suppression of KLF4 expression

**Figure 4:**
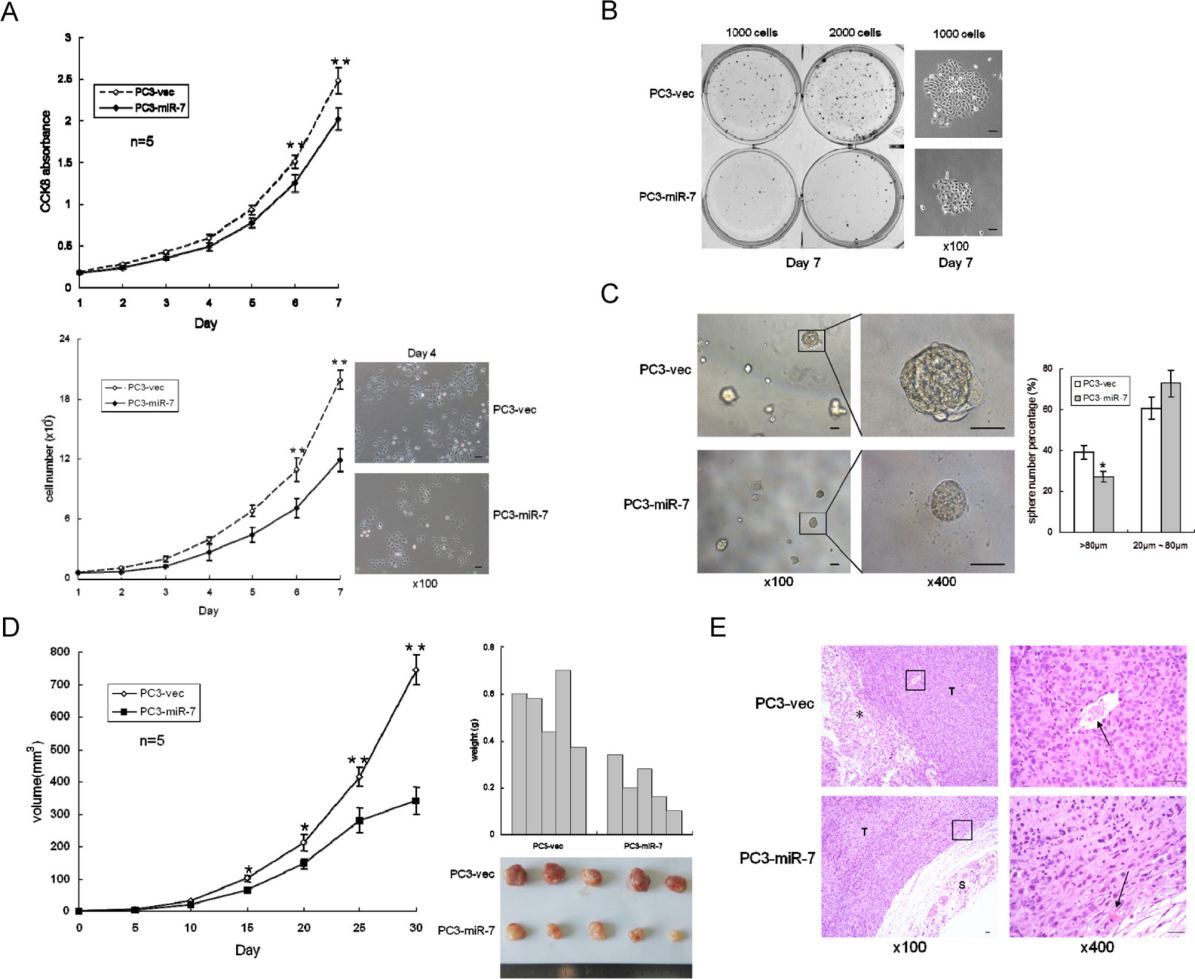
MiR-7 inhibits prostatic tumorigenesis **A.** Restoration of miR-7 inhibits cell proliferation by CCK8 test and cell number count. **B.** Restoration of miR-7 inhibits cell proliferation in 2-D colony formation. Magnification: ×100, Bar: 50 μm. **C.** Restoration of miR-7 inhibits 3-D sphere formation. Magnification: ×100; ×400, Bar: 50 μm. **D.** Restoration of miR-7 represses tumorigenesis *in vivo*. **E.** Restoration of miR-7 inhibits tumor growth in PC3-miR-7 derived xenograft. Black arrow: tumor vessel. T: tumor, S: skin. hexagram marker: adipose cells Magnification: ×100; ×400, Bar: 20 μm. Data are represented as mean ± SEM. *:*p* < 0.05; **:*p* < 0.01

Given the observation that miR-7 impaired the sphere formation and tumorigenesis in both stem-like and non stem-like cell derived grafts *in vitro* and *in vivo* (Figure 1D and 1E, Supplementary Figure 2), we speculated that the function of miR-7 on impairing PCSCs' stemness mainly, if not all, led to an overall inhibition on prostatic tumor growth. To assess the tumor suppressive function of miR-7 *in vitro*, cell proliferation were assessed. CCK8 test (a well-used assay for detection of cell proliferation and cytotoxicity [18, 19]) and cell number counting results showed that there was a significant inhibition of proliferation on PC3-miR-7 cells compared to PC3-vec cells (Figure 4A). We also found that miR-7 restoration significantly inhibited 2-D colony formation (Figure 4B) and 3-D sphere formation (Figure 4C) *in vitro*. We further compared the subcutaneous tumorigenesis of PC3-miR-7 vs PC3-vec derived grafts *in vivo* (Figure 4D). We found that tumor volumes and weights were significantly decreased by miR-7 restoration, indicating its tumor suppressive function in PCa. H&E staining also showed that miR-7 restoration inhibited tumor growth (Figure 4E). In order to confirm whether the inhibition by miR-7 restoration was mediated via specific reduction of KLF4 expression, we carried out KLF4 rescue assay. We found that cell proliferation was recovered in PC3-miR-7-KLF4 cells compared to the control (Supplementary Figure 6A). The suppression of 2-D colony formation and 3-D sphere formation was also impaired after overexpression of KLF4 (Supplementary Figure 6B and 6C). We also investigated whether KLF4 overexpression could recover the subcutaneous tumorigenesis that inhibited by miR-7 restoration (Supplementary Figure 6D). We found that tumor volumes and weights were significantly increased and cell proliferation was recovered by KLF4 rescue (Supplementary Figure 6D and 6E). All these data indicated that inhibition of overall prostatic tumor growth by miR-7 was achieved via suppression of KLF4. We further repeated the above experiments in PC3-shKLF4 vs PC3-con cells (Supplementary Figure 7). Our data showed that knock-down of KLF4 directly inhibited cell proliferation (Supplementary Figure 7A), 2-D colony formation (Supplementary Figure 7B) and 3-D sphere formation (Supplementary Figure 7C) *in vitro*, and subcutaneous tumorigenesis *in vivo* (Supplementary Figure 7D and 7E). Taken together, all findings above demonstrated that miR-7 restoration inhibits overall prostatic tumor growth *in vitro* and *in vivo* mainly due to suppression of the stemness factor KLF4.

### MiR-7-KLF4 axis inhibits overall prostatic tumor growth through PI3K/Akt pathway

**Figure 5:**
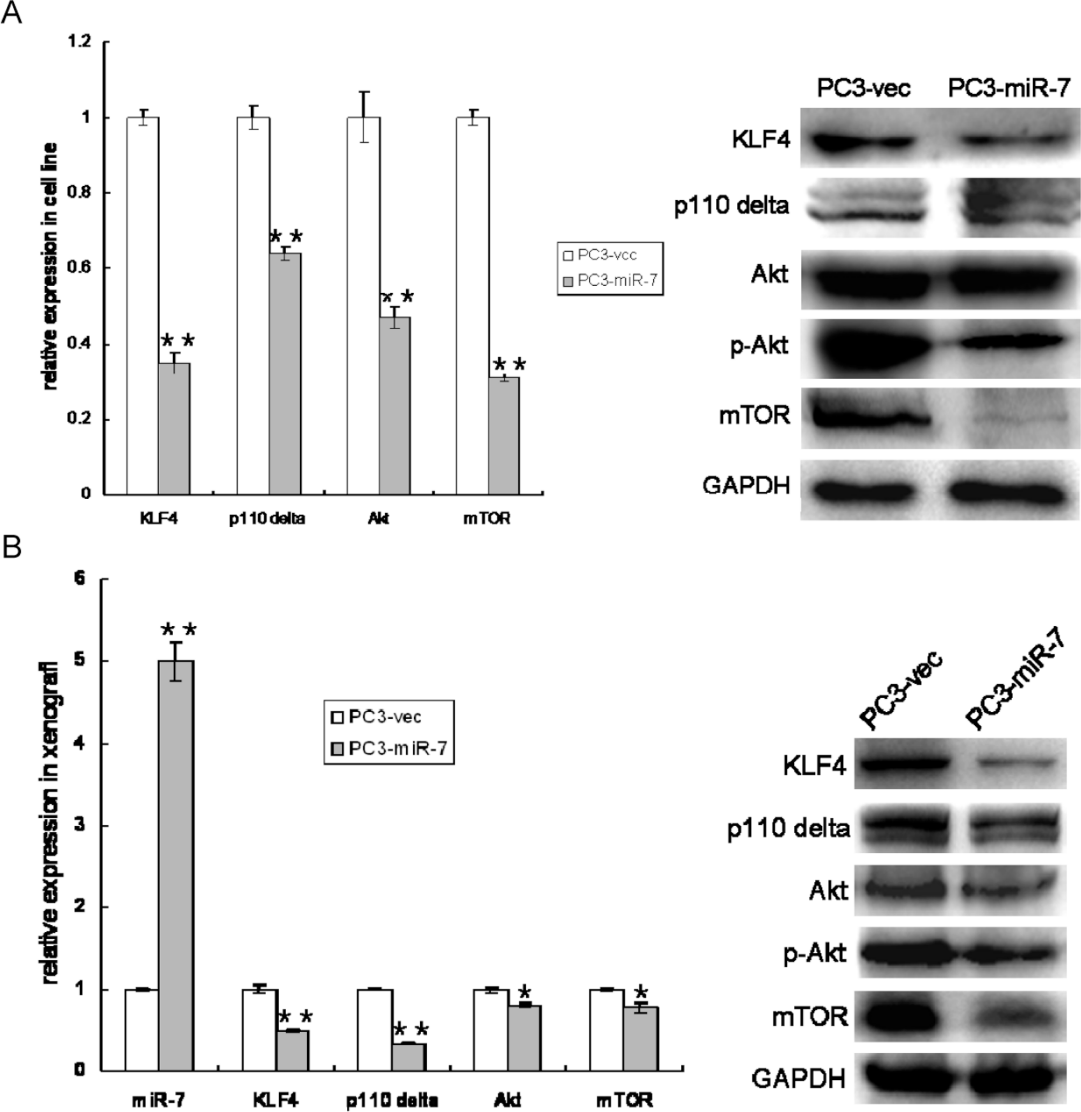
Restoration of miR-7 down regulates PI3K/Akt pathway which is mediated by KLF4 **A.** Restoration of miR-7 suppresses expression of PI3K/Akt pathway and KLF4 *in vitro*. **B.** MiR-7 sustains overexpression in PC3-miR-7 derived graft and inhibits expression of both KLF4 and PI3K/Akt pathway *in vivo*. Data are represented as mean ± SEM. *:*p* < 0.05; **:*p* < 0.01

Our previous finding [9] demonstrated that miR-7 inhibits proliferation and metastasis in liver cancer by suppressing PI3K/Akt pathway. Therefore, we further determined whether miR-7 restoration inhibits overall prostatic tumor growth also through attenuation of PI3K/Akt pathway and whether such attenuation is mediated by KLF4. We found that along with KLF4 suppression, p110δ, a catalytic subunit of PI3K in cancer [20], Akt and mTOR (another identified target of miR-7 [9]) were significantly down-regulated in PC3-miR-7 cells and its derived xenografts (Figure 5A and 5B). These findings indicated that PI3K/Akt pathway was indeed suppressed by miR-7 in PCa and in turn caused an overall inhibition of prostatic tumor growth. In order to confirm whether attenuation of PI3K/Akt pathway by miR-7 is mediated by KLF4, we checked the expression of p110δ, Akt and mTOR again after KLF4 rescue. We found that both mRNA and protein expressional levels of these genes in PC3-miR-7-KLF4 cells could be improved to the level that similar with PC3-vec cells. This result again indicated that suppression of PI3K/Akt pathway by miR-7 worked via inhibition of KLF4 (Supplementary Figure 8A and 8B). We further repeated above analyses in PC3-shKLF4 vs PC3-con cell lines and related derived xenografts (Supplementary Figure 9A and 9B). These analyses showed that the expression of PI3K/Akt pathway was significantly down-regulated after KLF4 knock-down directly in both PC3-shKLF4 cells and its derived grafts (Supplementary Figure 9A and 9B), indicating that KLF4 knock-down impaired the expression of PI3K/Akt pathway. As a critical transcription factor, KLF4 has been reported to activate transcription of multiple genes [21]. Combining the above data and the findings in other previous reports [22], we speculated whether KLF4 regulated the transcription of some components in PI3K/Akt pathway. By bioinformatics analysis (PROMO soft, http://alggen.lsi.upc.es) and chromatin immunoprecipitation (ChIP) sequencing, we observed that there are four KLF4 binding sites in the promoter region of p110δ. As shown in Supplementary Figure 9C, KLF4 was especially enriched at the 2^nd^ binding site (−824∼−820bp upstream of transcription start site) of p110δ promoter, demonstrating that KLF4 activated p110δ expression at transcriptional level. Thus our findings demonstrated that miR-7 inhibits overall prostatic tumor growth by suppression of KLF4/PI3K/Akt pathway.

### Restoration of miR-7 induces cell cycle arrest by increasing nuclear localization of p21, which is downstream of KLF4/PI3K/Akt axis

**Figure 6:**
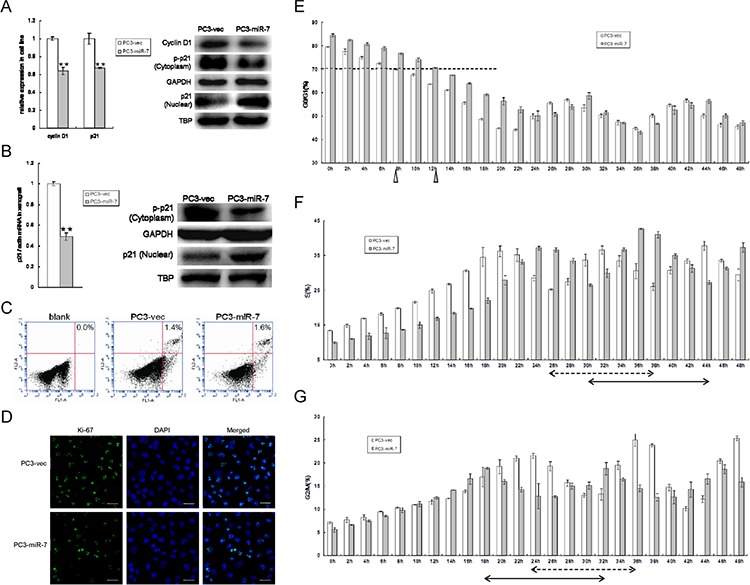
Restoration of miR-7 induces cell cycle arrest via increasing nuclear localization of p21 through suppression of KLF4/PI3K/Akt pathway **A.** Restoration of miR-7 suppresses total mRNA expression of p21 and cyclin D1, but decreases phosphorylation of p21 and increases nuclear localization of p21, which results in a predicative G0/G1 phase arrest in PCa *in vitro*. **B.** Restoration of miR-7 inhibits p21 total mRNA expression, but decreases phosphorylation of p21 and increases nuclear localization of p21 *in vivo*. **C.** No significant apoptosis occurs when restoration of miR-7 in PC3. **D.** Restoration of miR-7 suppresses expression of Ki-67 which indicates a predictive cell cycle arrest in PCa. Magnification: ×200, Bar: 20 μm. **E.** MiR-7 delays the restoration from serum starvation and induces G0/G1 arrest in PCa. White triangle: 8 hr is necessary for PC3-vec cells to recover growth, Gray triangle: 12 hr is necessary for PC3-miR-7 cells to recover growth. **F.** and **G.** Restoration of miR-7 delays cell cycle for about 2 hrs reflected by proportion change of cell number in either S phase (F) or G2/M phase (G) in PCa. Arrow with broken line: 12 hrs are needed for a cell cycle in PC3-vec cells. Arrow with full line: 14 hrs are needed for a cell cycle in PC3-miR-7 cells. TBP: TATA-binding protein, an internal control for nuclear protein. Data are represented as mean ± SEM. **:*p* < 0.01

Given that restoration of miR-7 abrogates KLF4/PI3K/Akt pathway and eventually inhibits prostate tumorigenesis, we attempted to identify additional downstream cascade effectors of the signaling pathway. We paid particular attention to p21, a well-known inhibitor of cell cycle progression and a key effecter of multiple pathways including p53/KLF4 pathway [23]. As studies reported that phosphorylation of p21 by Akt activation keeps it in the cytoplasm for anti-apoptosis while non-phosphorylated p21 shuttled into the nucleus for G1-S phase arrest [24], we wondered whether suppression of KLF4/PI3K/Akt pathway by miR-7 restoration inhibits prostate tumorigenesis through p21. We assessed the expression and phosphorylation of p21 and found that restoration of miR-7 reduced p21 expression and cyclin D1, a cell cycle activator at mRNA level (Figure 6A). We further checked p21 phosphorylation levels in PC3-miR-7 vs PC3-vec cells. We found that phosphorylated p21 in the cytoplasm was decreased upon the dramatically inhibition of Akt phosphorylation by miR-7 restoration (Figure 5A and 5B), while nuclear localization of p21 was increased though the total expression of p21 was down-regulated (Figure 6A). We repeated the same assay in PC3-miR-7 vs PC3-vec derived grafts and again observed increased nuclear localization of p21 (Figure 6B). In addition, we observed that KLF4 rescue enhanced the expression of cyclinD1 and phosphorylation of p21 and in turn decreased the nuclear localization of p21 (Supplementary Figure 10A and 10B). In addition, we also observed that KLF4 knock-down directly accumulated p21 in the nucleus both *in vitro* and *in vivo* (Supplementary Figure 11A and 11B). Thus, miR-7 restoration stimulated the nuclear localization of p21 by reducing its phosphorylation through KLF4/PI3K/Akt pathway. In addition, we performed Ki-67 staining and apoptosis assays to study possible effects of p21 on cell cycle arrest and apoptosis respectively. We found that while Ki-67 staining was obviously reduced by miR-7 restoration, there was no significant difference of apoptosis between PC3-miR-7 and PC3-vec cells *in vitro* (Figure 6C) indicating a potential cell cycle arrest (Figure 6D). We then carried out a 48 hrs continuous observation on cell cycle progression in PC3-miR-7 vs PC3-vec cells. After 36 hrs serum starvation for synchronization, it took about 12 hrs for PC3-miR-7 cells to recover growth (G0/G1 < 70%, Figure 6E), while 8 hrs was sufficient for PC3-vec cells, which indicated that miR-7 restoration prevented cell from activation. By comparing the proportion change in S and G2/M phase, we found that PC3-miR-7 cells needed about 14 hrs to complete a cell cycle progression but PC3-vec cells only needed 12 hrs, which indicated a 2 hrs cell cycle delay by miR-7 restoration (Figure 6F and 6G). Thus our data confirmed the cell cycle arrest function of miR-7 via nuclear increase of p21 through the miR-7/KLF4/PI3K/Akt pathway. All the above findings together demonstrated that miR-7 restoration impairs the stemness of PCSCs and inhibits prostate tumorigenesis via the KLF4/PI3K/Akt/p21 axis.

### MiR-7 and KLF4 expression is associated with tumor progression in clinical samples

**Figure 7:**
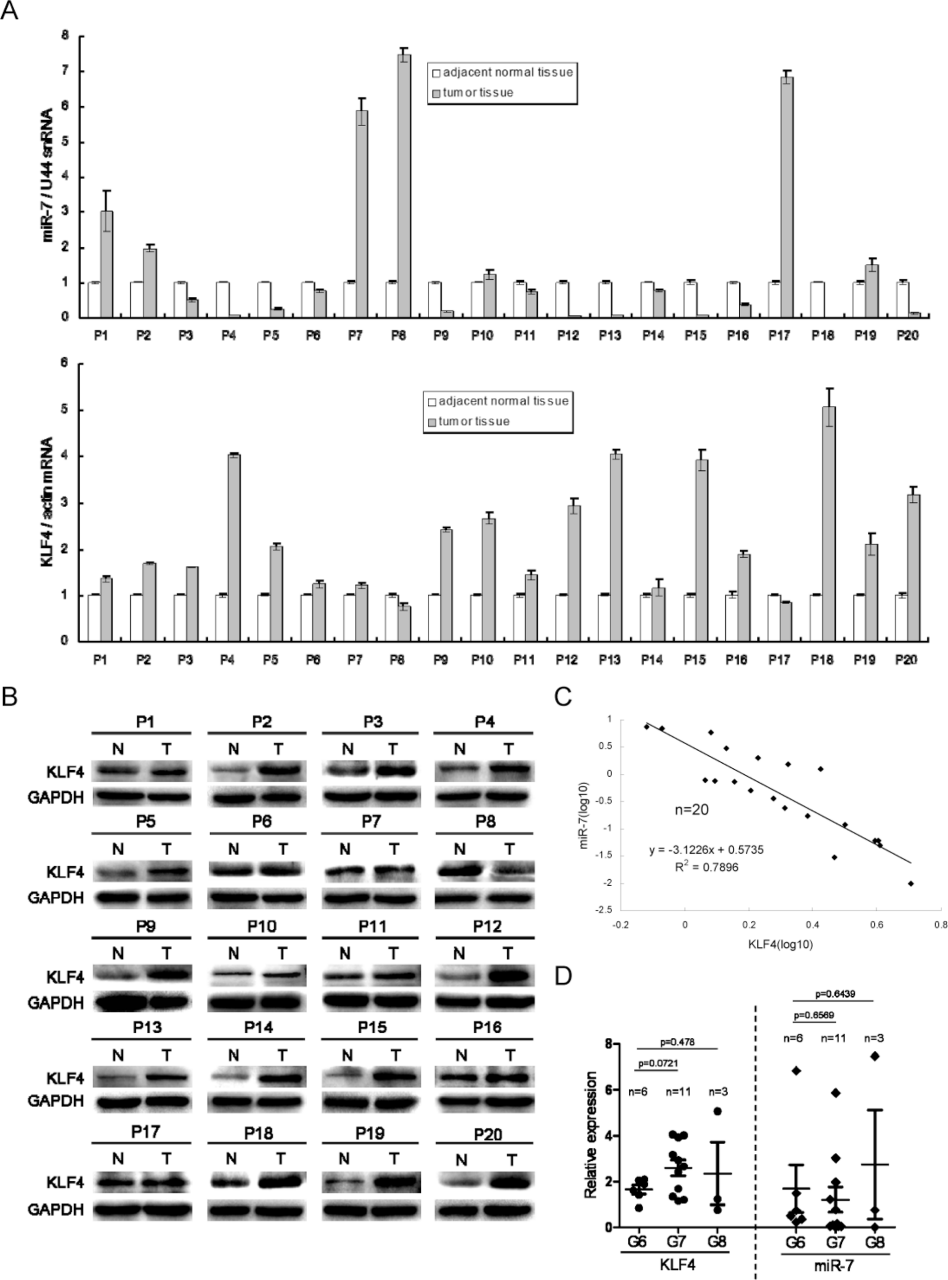
MiR-7 expression is negatively correlated with KLF4 and is associated with tumor progression in clinical samples **A.** MiR-7 is decreased but KLF4 mRNA is increased in most samples of 20 patients. **B.** KLF4 expression is increased in patient samples at protein level. N: adjacent normal tissue, T: tumor tissue. **C.** KLF4 expression is negatively correlated with miR-7 in patient samples. **D.** KLF4 is positively while miR-7 is negatively associated with PCa progression by Gleason score evaluation. G6/G7/G8: Gleason score 6/7/8. Data are represented as mean ± SEM.

Given the above findings in xenograft animal models, we next investigated whether KLF4 expression was negatively correlated with miR-7 levels in tumor tissues and its relationship with tumor progression. We compared KLF4 and miR-7 expression in tumors and paired adjacent normal tissues in 20 patients (Figure 7A and 7B). We found that KLF4 was up-regulated in 90% tumor samples (18/20), and miR-7 was down-regulated in 65% tumor samples (13/20). Among the tumor tissues in which miR-7 was suppressed, KLF4 expression was significantly increased than paired adjacent normal tissues. Correlation analysis by Pearson's chi-square test indicated that KLF4 expression was negatively correlated with miR-7 expression in these 13 paired specimens (r^2^= 0.7896, Figure 7C). Though there was no statistical significance among the samples with various Gleason scores potentially due to limited sample numbers, the KLF4 expression trended to be increased (1.66 ± 0.19 vs 2.60 ± 0.34, Gleason score 6 vs Gleason score 7) while miR-7 expression trended to be decreased (1.7 ± 1.04 vs 1.21 ± 0.55, Gleason score 6 vs Gleason score 7) along with the increase of Gleason score (Figure 7D, Supplementary Table 1). These results indicated that KLF4 expression was positively while miR-7 expression was negatively correlated with tumor progression.

## DISCUSSION

Although CSC theory has received great attention and eradication of CSCs is generally believed more effective for arresting tumor progression and reversing therapeutic resistance, the regulatory mechanisms of CSCs are still poorly understood. In the present study we identified miR-7 as a novel miRNA to specifically suppress the PCSCs' stemness and prostate tumorigenesis by directly inhibiting a key stemness factor KLF4. Furthermore, we also demonstrated that miR-7 restoration inhibits overall prostate tumorigenesis through KLF4/PI3K/Akt/p21 pathway (Supplementary Figure 12). Importantly, we found that miR-7 expression is negatively correlated with KLF4 expression in clinical samples and is reversely associated with Gleason Scores during PCa progression, indicating the importance of miR-7 expression in human PCa pathology and treatment.

Compared to previous studies on miRNAs in PCSCs [25, 26], the current study demonstrates a different mechanism. For example, restoration of miR-34a in PCa inhibits PCSC characteristics via suppressing stemness-related surface marker CD44 expression [25]. Restoration of miR-200 inhibits PCSCs' stemness indirectly via suppressing Zeb1/2 and Snial2 expression and reversing EMT [26]. Here we observed a new mechanism of miR-7′s inhibitory function on PCSCs' stemness by directly suppressing KLF4 expression at post-transcriptional level. In addition, compared with miR-34a and miR-200, our data show that down-regulation of miR-7 is more significant in CD44+CD133+ [16] stem-like cells sorted from PC3 derived grafts (Supplementary Figure 13A). Thus, our study not only adds miR-7 to the existing list of specific miRNAs to suppress the PCSCs' stemness, but also indicates a direct correlation between miR-7 and a specific stemness factor KLF4. By suppression of KLF4 and its downstream PI3K/Akt/p21, miR-7 impairs PCSCs' stemness and overall prostate tumor growth.

Furthermore it is important to note that the inhibitory function by miR-7 restoration on PCSCs' stemness can be sustained for generations. In our study, we compared KLF4 expression in stem-like cells sorted from PC3-miR-7 vs PC3-vec derived parental (g0), 1^st^ (g1) and 2^nd^ generation (g2) grafts respectively (Supplementary Figure 13B). We found that miR-7 restoration continuously suppressed KLF4 expression in all the generations (Supplementary Figure 13B). Functionally, restoration of miR-7 significantly reduces sphere formation *in vitro* and tumorigenesis *in vivo* in grafts of all the three generations. Meanwhile, after non stem-like cells were sorted from either PC3-vec or PC3-miR-7 derived grafts and inoculated for forming the next generation grafts, stem-like cells can again be sorted from these non stem-like cells derived grafts. Even in these non stem-like cell derived stem-like cells restoration of miR-7 still suppressed KLF4 expression and impaired sphere formation and tumorigenesis *in vitro* and *in vivo* respectively.

Consistent with our previous work that miR-7 inhibited proliferation and metastasis in liver cancer by suppressing PI3K/Akt pathway [9], the present study show that miR-7 also works as a tumor suppressor in PCa by attenuating PI3K/Akt signaling. More importantly, we provided evidence that KLF4 down-regulation suppressed the transcription of p110δ, a major catalytic subunit of PI3K in cancer cells [20], so to directly reduce PI3K/Akt pathway activity and eventually suppressed the phosphorylation of both Akt and p21 and stimulated the nuclear translocation of p21. Together, these findings demonstrated that restoration of miR-7 impaired the PCSCs' stemness and prostate tumorigenesis by suppressing KLF4/PI3K/Akt/p21 signaling. This study sheds new lights on our understanding of molecular pathogenesis of PCa and implicates that miR-7 might have potential applications for PCa prognosis and treatment.

## METERIALS AND METHODS

### Ethics statement

Investigation has been conducted in accordance with the ethical standards and according to the Declaration of Helsinki and according to national and international guidelines and has been approved by the authors' institutional review board.

### Cell lines and cell culture

Normal human prostate epithelial cell line RWPE1, BPH-1 and PCa cell lines PC3, DU145, LNCaP were obtained from the American Type Culture Collection (ATCC). All the cells were cultured in RPMI 1640 basic medium (Invitrogen, Carlsbad, CA, USA) with 10% fetal bovine serum (Gibco, Grand Island, NY, USA) and maintained at 5% CO_2_ at 37°C.

### Plasmid construction

The backbone vector pEGP-miR-null (Cell Biolabs, San Diego, CA, USA) was double-digested with BamHI/NheI to insert human pri-miR-7–1 fragment (Supplementary List 1) and was re-named pEGP-miR-7. pGFP-V-RS vector for KLF4 knock down (TG316853) and its control (TR30013) were purchased from Origene (Rockville, MD, USA).

A firefly luciferase expressional vector phE-luc [27] was employed as backbone for inserting an artificial miR-7 target sequence (Supplementary List 2) to construct the positive control phE-luc/miR-7-target. Full length KLF4 3′UTR was amplified using primers: 5′-GTTGCTAGCATATGACCCACACTGCCAGA-3′, 5′- GTCGATATCGTGCGTGCTTCTTACATGCC-3′. Wide type (WT) and mutant (MT) sequences of two putative miR-7 target sites (A and B) and an artificial target C (an integration of A and B, Figure 3) were synthesized (Sangon Biotech Comp, Shanghai, China) respectively. All the sequence were double-digested with EcoRV/NheI, sequentially inserted into phEW-luc/miR-7-target for replacing the positive control sequence to generate relevant luciferase report vectors, named as phE-luc/KLF4-A-WT, phE-luc/KLF4-A-MT, phE-luc/KLF4-B-WT, phE-luc/KLF4-B-MT, phE-luc/KLF4-C-WT, phE-luc/KLF4-C-MT, phE-luc/KLF4-FL, respectively. All the restriction endonucleases were purchased from New England Biolabs (Ipswich, MA, USA).

### Cell transfection and luciferase reporter assays

All vectors were transfected with Lipofectamine 2000 (Invitrogen). Puromycin was used for selecting subclones stably expressing miR-7 or KLF4-shRNA.

For luciferase assay, 10^5^/well PC3 cells in 24-well plates were co-transfected with 200ng reporter vectors, 5ng pRL-CMV (internal standard, Promega, Madison, WI, USA), and 5 nM miR-7 precursor or scrambled control (Ambion, Carlsbad, CA, USA) using Lipofectamine 2000 (Invitrogen). 24 hrs after trasfection, luciferase activities were measured with the Dual-Luciferase Reporter Assay System (Promega). Reporter luciferase activity was normalized to the internal control Renilla luciferase activity in all samples.

### Lentivirus infection

Lentivirus for stable expression of KLF4 coding sequence (without 3′UTR) or control was constructed and identified by Genomeditech Comp. (Genomeditech, Shanghai, China). 2 × 10^6^ cells were seeded in 6-well plates and infected using relevant lentivirus (MOI = 10 for each) concomitant with 5 μg/ml polybrene. qRT-PCR and western blot were employed to detect KLF4 expression levels 36 hours after infection. MOI: multiplicity of infection.

### Cell proliferation

Cell proliferation was measured by cell number counting and CCK-8 assay [18, 19] (Dojindo, Kumamoto, Japan). 5 × 10^3^ cells/well was seeded in 24-well plates. Total cell numbers were counted daily for 1 week after seeding. For CCK-8 assay, cells were incubated with CCK-8 for 2 hrs at 37°C, and detected the absorbance at 450 nm.

### Cell cycle analysis

10^5^ cells per well were seeded in 6-well plate and were first synchronized by serum starvation for 36 hrs and stained with hoechest (Molecular Probes, Eugene, OR, USA) for 10 min at room temperature at relevant time points for flow cytometry and analyzed using Modifit analysis (Beckman Coulter, Brea, CA, USA) software.

### 3D-sphere formation

200 cells per well were seeded in 96-well plate and were coated with solidified mixture (1:1) of geltrex (Gibco) and matrigel (BD Biosciences, Bedford, MA, USA). Two weeks after plating, spheres were counted and imaged.

### 2D-colony formation

One week after seeding cells in 6-well plate, formed colonies were stained with 0.1% crystal violet (Sigma-Aldrich, St. Louis, MO, USA) for imaging and counting.

### qRT-PCR

Total RNA was extracted using TRIzol (Invitrogen) according to the protocol previously described [9]. MiRNA was extracted using miRNA isolation kit (Ambion). MiRNA reverse transcription and qRT-PCR were carried out using Taqman miRNA reverse transcription kit (Applied Bio-systems, Carlsbad, CA, USA) and Taqman premix (Takara, Shiga, Japan) respectively. The specific reverse primers and qRT-PCR Taqman probes for miR-7, miR-34a, miR-200a and snRNA U44 (internal normalization control) were purchased from Applied Bio-systems. For mRNA analysis, total RNA was reversed transcribed with Prime-Script RT kit (Takara), and amplified with SYBR Green Real-time PCR Master Mix (Applied Bio-systems). The mRNA level of Actin was used as internal normalization control. All primers are available in the Supplementary List 3.

### Western blot

Total protein extractions were prepared, subjected to 10% sodium dodecyl sulfate polyacrylamide gel (SDS-PAGE) electrophoresis, transferred onto nitrocellulose membranes and probed with primary and in turn secondary antibodies (Supplementary List 4) according to the methods described previously [9]. Specific proteins were detected with Immobilon Western Chemiluminescent HRP Substrate (Millipore, Billerica, MA, USA).

### Immunofluorescent staining

Cells were fixed with 4% formaldehyde and incubated with relevant primary antibodies (Supplementary List 4) for 16 hrs at 4°C, followed by secondary antibody for detecting. Nuclei were stained with DAPI (Sigma-Aldrich).

### Flow cytometry

Cells were harvested from xenografts by collagenase IV (Abcam, Eugene, OR, USA) and 0.05% trypsin (Sigma-Aldrich) digestion, and incubated with conjugated antibodies (Supplementary List 4) for flow cytometry assay.

### ChIP assay

ChIP assay was performed using Chromatin IP kit (Cell Signaling Technology, Beverly, MA, USA) according to the manufacturer's protocol. Briefly, 10^7^ adherent PC3 cells were crosslinked with formaldehyde, collected and digested to produce chromatin fragments. KLF4 antibodies (Santa Cruz, Dallas, TX, USA) and normal goat IgG control (Cell Signaling Technology) were used for ChIP respectively. ChIP DNA was purified and analyzed by qPCR and sequencing. KLF4 relative enrichment was calculated by the formula provided in the protocol. ChIP primers are available in the Supplementary List 5.

### Apoptosis assay

Apoptosis assay was processed with Annexin V-FITC Kit (Beckman Coulter), according to the manufacturer's protocol. Briefly, 10^5^ cells were collected and dual-incubated with Annexin V-FITC and PI for flow cytometry assay.

### Animal experiments

5-week-old male BALB/C athymic nude mice (SLAC, Shanghai, China) were housed and manipulated according to the protocols approved by the Renji Hospital Medical Experimental Animal Care Commission. Xenograft volume was calculated per 5 days for 30 days according to the formula: width × length × height × π/2. After mice were sacrificed, xenografts were collected for RNA and protein extraction and H&E staining.

### Clinical samples

Frozen PCa and paired adjacent normal tissues were obtained from Renji Biobank, Shanghai Jiaotong University School of medicine [28] (Shanghai, China). Written informed consent was obtained from all patients. The studies using human tissues were reviewed and approved by the Committee for Ethical Review of Research Involving Human Subjects at Renji Hospital.

### Statistical analysis

Independent Student's *t*-test and analysis of variance (ANOVA) were used for comparisons of differences between two groups. The correlation between miR-7 and KLF4 mRNA expression was evaluated using Pearson's chi-square test. Relationship between KLF4 or miR-7 expression with Gleason Score was analyzed using Prism GraphPad 5 (GraphPad Software, La Jolla, CA, USA). All data were represented as mean ± SEM from triplicate experiments. Results were considered statistically significant at *p* < 0.05.

## SUPPLEMENTARY MATERIALS LIST


