# Supplementary material for: MicroRNA-7 inhibits the stemness of prostate cancer stem-like cells and tumorigenesis by repressing KLF4/PI3K/Akt/p21 pathway
Source: Oncotarget. 2015 Jun 29;6(27):24017–31. doi: 10.18632/oncotarget.4447 (PMC4695167; doi:10.18632/oncotarget.4447)
Supplement: Supplementary file 1 [file oncotarget-06-24017-s001.pdf]

SUPPLEMENTARY MATERIALS LIST

List 1: Human pri-miR-7–1 sequence

Synthesized sequence (241bp) containing human pri-miR-7-1 (NR_029605.1) fragment (red):

5′- taggatccgg tgaaaactgc tgccaaaacc acttgttaaa aattgtacag agcctgtaga aaatatagaa gattcattgg atgttggcct agttctgtgt ggaagactag tgattttgtt gtttttagat aactaaatcg acaacaaatc acagtctgcc atatggcaca ggccatgcct ctacaggaca aatgattggt gctgtaaaat gcagcatttc acaccttact agctctagac g -3′

In this sequence, "ggatcc" (underlined) is for BamHI digestion, "tctaga" (underlined) is for XbaI (an isocaudarner of NheI, which is located in the backbone vector) digestion. Full-length pri-miR-7-1 sequence (110bp) is shown in red.

List 2: An artificial miR-7 target sequence completely matched with human miR-7 as a positive control

5′- GATCTGCTAGCCAACAAAATCACTAGTCTTCCAGATATCAGATCT -3′

In this sequence, "AGATCT" is for BglII digestion, "GCTAGC" is for NheI digestion and "GATATC" is for EcoRV digestion. Underlined sequence "TCTTCC" is the core binding sequence for miR-7. Both the sequence and phE-luc plasmid were digested by BglII to insert the sequence and the orientation was confirmed by sequencing. By NheI/EcoRV double-digestion, this positive control sequence could be replaced by either full-length KLF4 3′UTR or relevant target fragment described in the text.

List 3: Primers for qRT-PCR

KLF4: forward: 5′- TTACCAAGAGCTCATGCCAC -3′
reverse: 5′- TGTGCCTTGAGATGGGAACT -3′
Sox2: forward: 5′- ACCTACAGCATGTCCTACTC -3′
reverse: 5′- AGTGGGAGGAAGAGGTAAC -3′
OCT4: forward: 5′- CTCACTTCACTGCACTGTAC -3′
reverse: 5′- GTTTGAATGCATGGGAGAGC -3′
Nanog: forward: 5′- AGCCAAATTCTCCTGCCAGT -3′
reverse: 5′- CACGTCTTCAGGTTGCATGT -3′
p21: forward: 5′- AAGACCATGTGGACCTGTCA -3′
reverse: 5′ - AATCTGTCATGCTGGTCTGC -3′
cyclin D1: forward: 5′- GTGCCACAGATGTGAAGTTC -3′
reverse: 5′ - CACACTTGATCACTCTGGAG -3′
p110δ: forward: 5′- TCGCCAACATCCAACTCAAC -3′
reverse: 5′- CACACAATAGCCAGCACAGG -3′
Akt: forward: 5′- CTTCTTTGCCGGTATCGTGT -3′
reverse: 5′- TGTCATCTTGGTCAGGTGGT -3′
mTOR: forward: 5′- AGGGTTCGAGATAAGCTCAC -3′
reverse: 5′- TAGCACTGGCAGAGGTTTTC -3′

List 4: Antibodies for Western Blot, Immunofluorescent Staining and Flow Cytometry Sorting

Antibodies for western blot:

KLF4, p110δ (Santa Cruz)
p21, p85, AKT, p-AKT, mTOR, p70S6K, TBP (Cell Signaling Technology)
GAPDH (Invitrogen)

Antibodies for immunofluorescent staining (IF):

Ki67 (Abcam, rabbit)
Sox2, OCT4, Nanog (Cell Signaling Technology, rabbit)
KLF4 (Santa Cruz, rabbit)
Alexa 594 donkey anti-rabbit (Invitrogen, secondary antibody)

Antibodies for flow cytometry sorting:

Mouse anti-human CD44-APC, mouse anti-human CD133-PE (Miltenyi Biotec, Bergisch Gladbach, Germany)

List 5: primers for ChIP qPCR

Binding site 1: forward: 5′- CACAGCTGGCTTCACACCC -3′
reverse: 5′- CTGCGCAGGGATTCAAACC -3′
Binding site 2: forward: 5′-ATTTTCAGCGGGTTCTCTCC -3′
reverse: 5′- ACAGCAGCCCCAGTCCTTAG -3′
Binding site 3–4: forward: 5′- GTCTAAAACAAAGCCCCTCG -3′
reverse: 5′- AAAACAGGAAGTGGAGAGCG -3′
Control: forward: 5′- CACCAGTTTGAGACCAGCCT -3′
reverse: 5′- CCTCCCAGATTCAAGCGATT -3′

SUPPLEMENTARY FIGURES AND TABLE

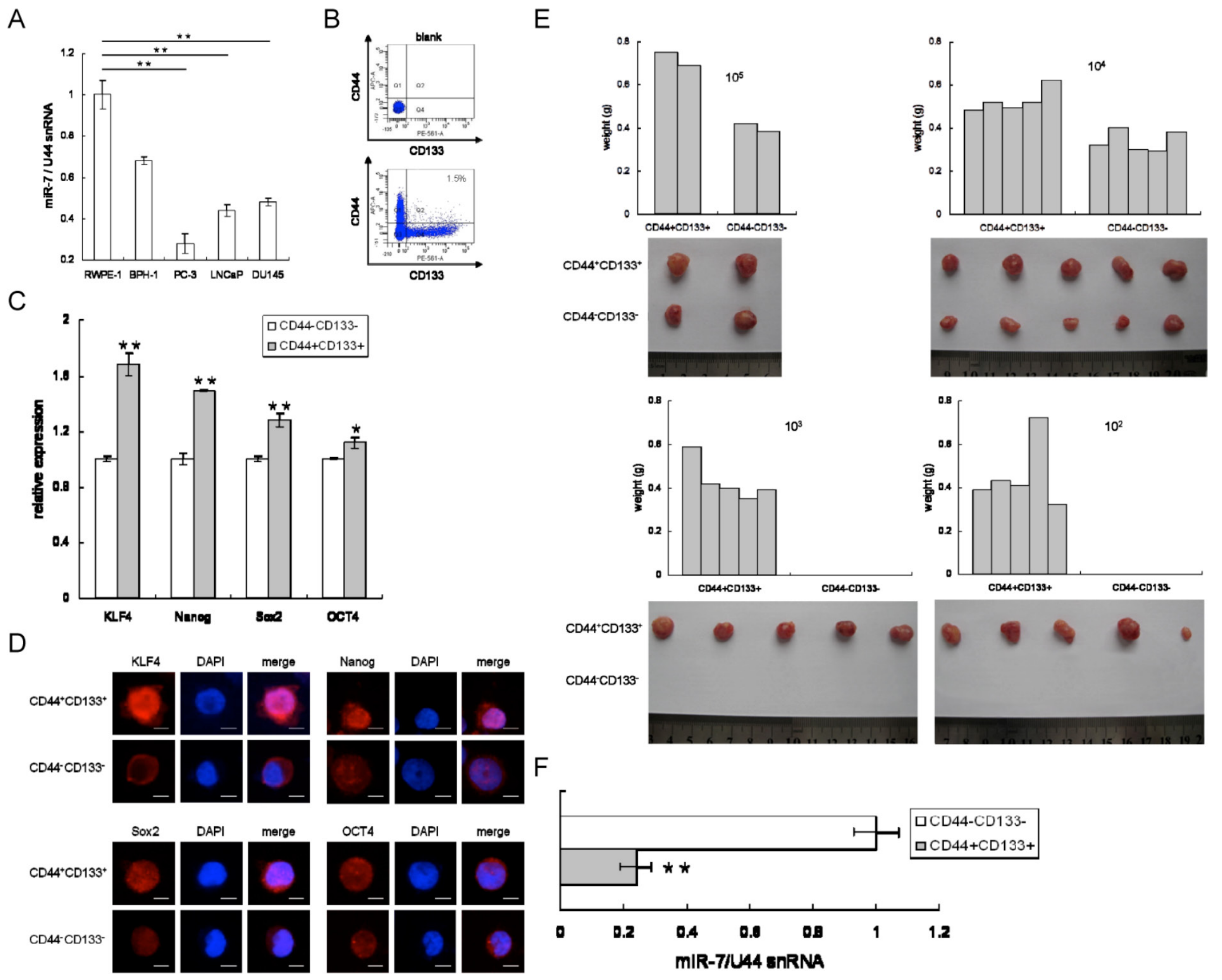

Supplementary Figure 1: MiR-7 is a tumor suppressor and is suppressed in CD44+CD133+ cancer stem-like cells in PCa. A. MiR-7 is significantly suppressed in PCa cell lines compared with normal cell line RWPE-1. **B.** CD44+CD133+ subpopulation is sorted from PC3 derived grafts by FACS. Blank: without antibody incubation. **C.** and **D.** The expression of stemness factors is increased in CD44+CD133+ subpopulation at both mRNA (C) and protein (D) levels. Magnification: ×200, Bar: 10 μm. **E.** Limited dilution assay demonstrates a stronger tumorigenicity of CD44+CD133+ subpopulation. **F.** The expression of miR-7 is significantly reduced in CD44+CD133+ subpopulation. Data are represented as mean ± SEM. *:$p < 0.05$; **:$p < 0.01$.

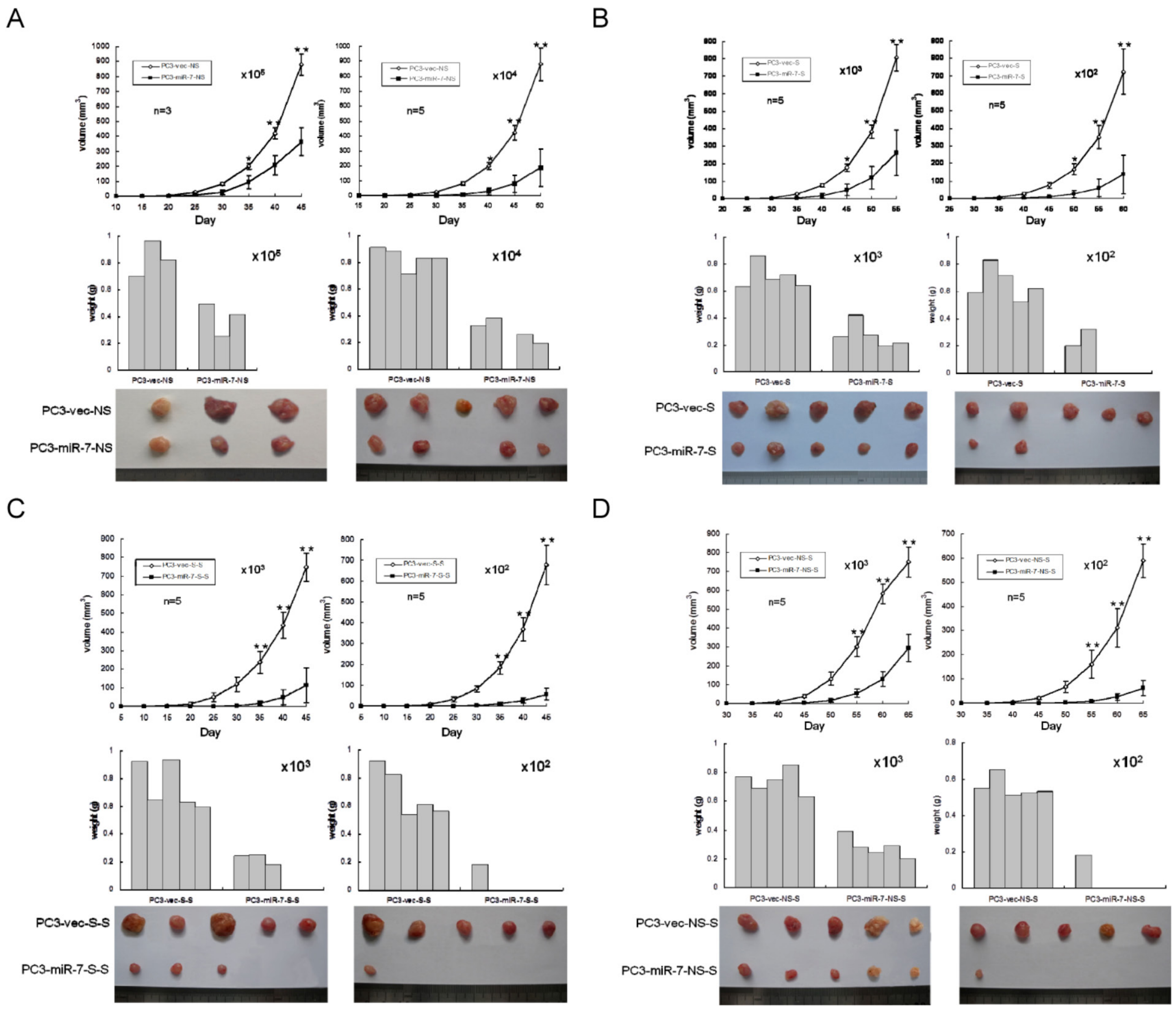

Supplementary Figure 2: Restoration of miR-7 impairs the tumorigenesis of both 1st generation (g1) and 2nd generation (g2) stem-like cells derived grafts. **A.** Restoration of miR-7 decreases the volume and weight of g1 non stem-like cells derived grafts using 10^5 or 10^4 cells for inoculation. **B.** Restoration of miR-7 decreases the volume and weight of g1 stem-like cells derived grafts using 10^3 or 10^2 cells for inoculation. **C.** Restoration of miR-7 decreases the volume and weight of g2 stem-like cells derived grafts using 10^3 or 10^2 cells for inoculation. These g2 stem-like cells are sorted from PC3-miR-7-S vs PC3-vec-S derived grafts. **D.** For those g2 stem-like cells sorted from PC3-miR-7-NS vs PC3-vec-NS derived grafts, restoration of miR-7 also decreases the volume and weight of relevant g2 grafts using 10^3 or 10^2 cells for inoculation. Data are represented as mean ± SEM. *:$p < 0.05$; **:$p < 0.01$.

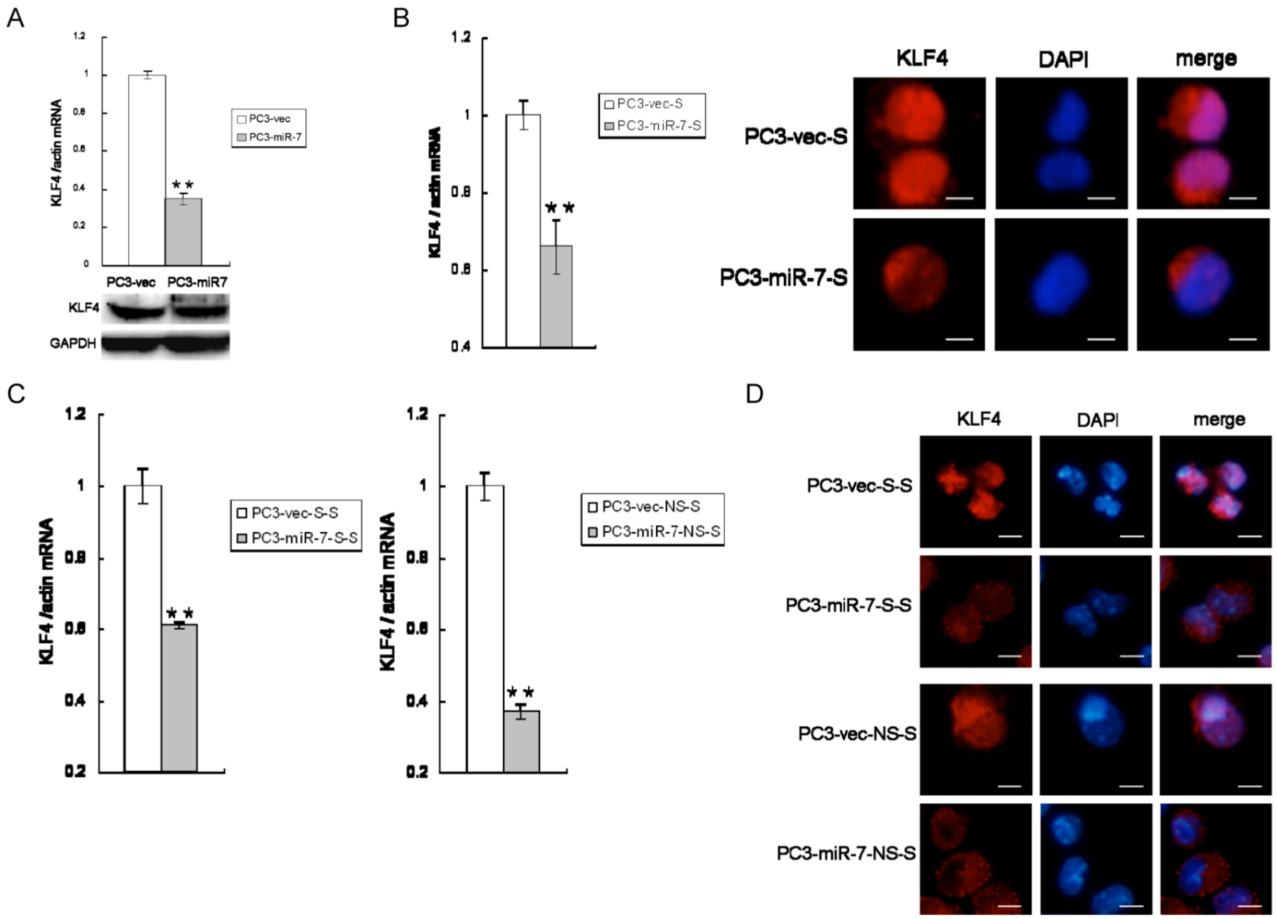

Supplementary Figure 3: KLF4 is continuously suppressed by miR-7 for generations. A. MiR-7 significantly decreases KLF4 expression in PC3-miR-7 subclone cell line at both mRNA and protein levels. **B.** Expression of KLF4 is decreased in stem-like cells sorted from PC3-miR-7 derived grafts. Magnification: ×200, Bar: 10 μm. **C.** and **D.** For those g2 stem-like cells sorted from PC3-miR-7-NS vs PC3-vec-NS derived grafts or PC3-miR-7-S vs PC3-vec-S derived grafts, restoration of miR-7 also inhibits KLF4 expression at both mRNA (C) and protein (D) levels. Magnification: ×200, Bar: 10 μm. Data are represented as mean ± SEM. **:$p < 0.01$.

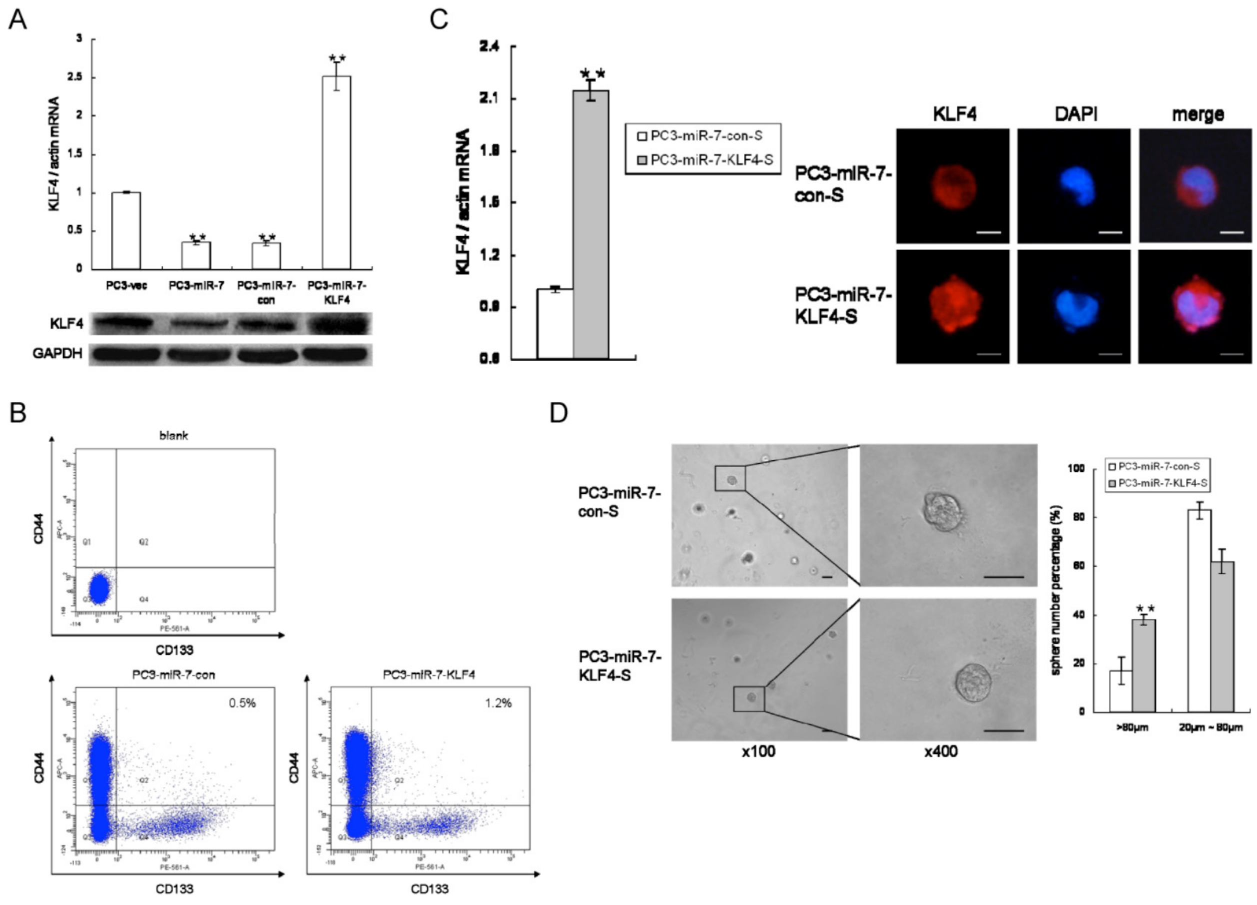

Supplementary Figure 4: Overexpression of KLF4 coding sequence rescues the stemness of PCSCs. A. KLF4 coding sequence (without 3′UTR) is stable overexpressed in PC3-miR-7 cells by lentivirus infection. **B.** Rescue of KLF4 increases the proportion of PCSCs sorted from PC3-miR-7-KLF4 cells compared to the control cells. **C.** KLF4 expression is improved in PC3-miR-7-KLF4 cells at both mRNA and protein levels. Magnification: ×200, Bar: 10 μm. **D.** Rescue of KLF4 recovers sphere formation of PC3-miR-7-KLF4-S cells *in vitro*. Magnification: ×100; ×400, Bar: 50 μm. Data are represented as mean ± SEM. **:$p < 0.01$.

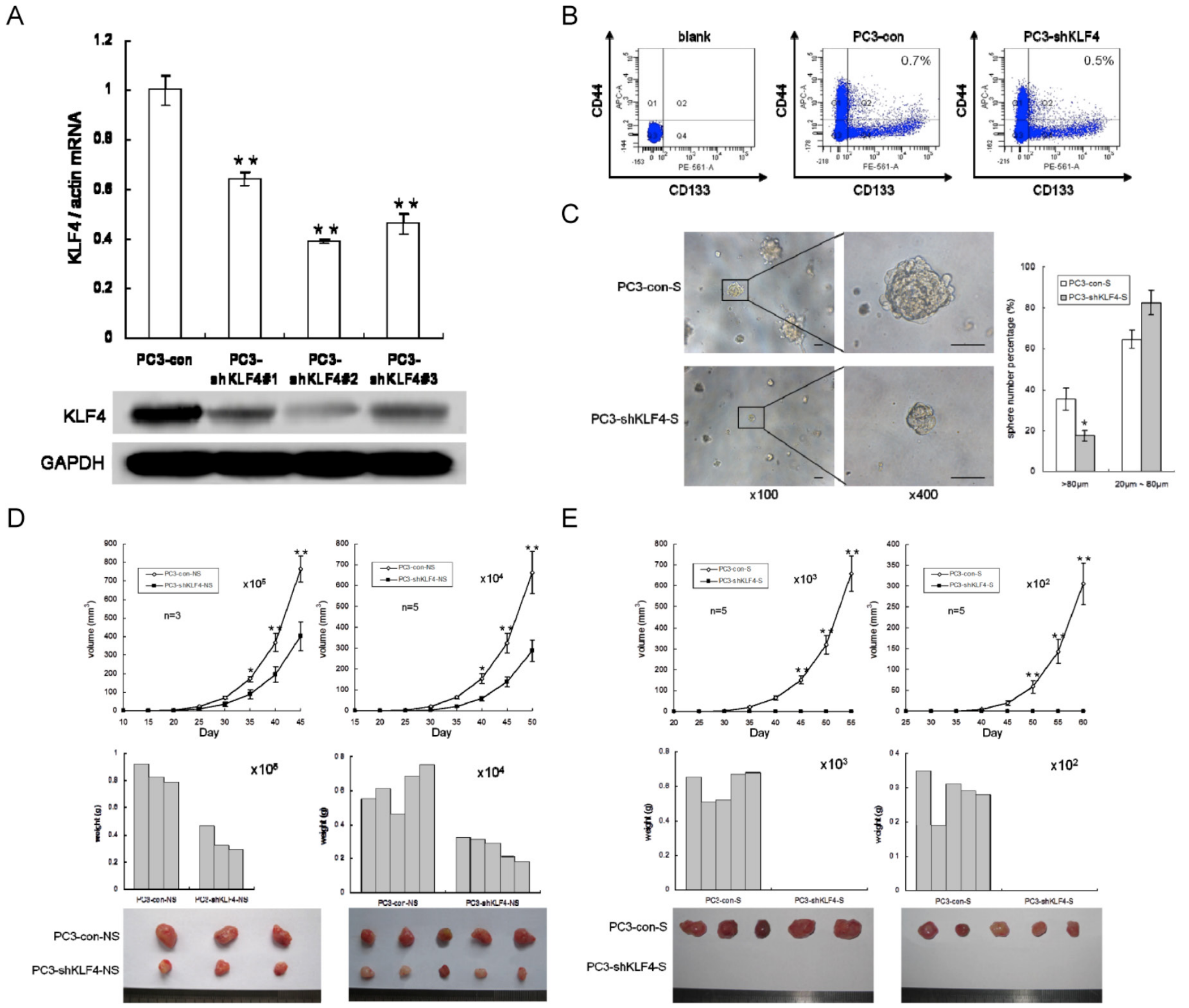

Supplementary Figure 5: Knock-down of KLF4 directly impairs the stemness of PCSCs. A. Expression of KLF4 is decreased at both mRNA and protein levels in three independent strains of KLF4 knock-down subclone cell lines by using relevant KLF4 shRNA. The second subclone showed most significant repression of KLF4 and is selected for all the following assays and re-named as PC3-shKLF4. **B.** Knock-down of KLF4 directly decreases the proportion of PCSCs compared to the control. PCSCs are sorted from PC3-shKLF4 and PC3-con derived grafts respectively. Blank: without antibody incubation. **C.** Knock-down of KLF4 directly inhibits sphere formation in PCSCs *in vitro*. Magnification: ×100; ×400, Bar: 50 µm. **D.** Knock-down of KLF4 directly decreases the volume and weight of non stem-like cell derived grafts using 10^5 or 10^4 cells for inoculation. **E.** Knock-down of KLF4 directly blocks the tumorigenesis of stem-like cells derived grafts completely using either 10^3 or 10^2 cells for inoculation. Data are represented as mean ± SEM. *:$p < 0.05$; **:$p < 0.01$.

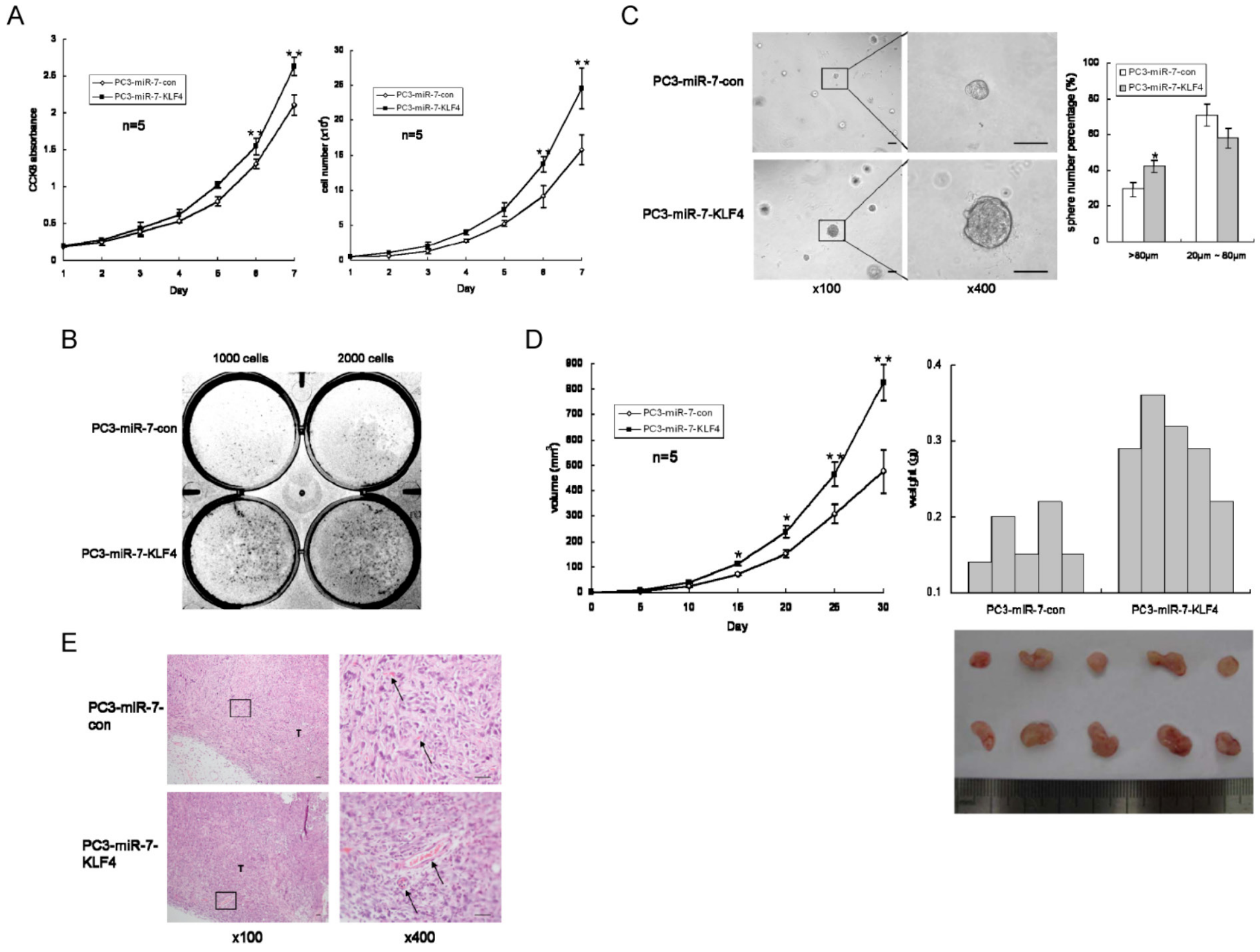

Supplementary Figure 6: Overexpression of KLF4 coding sequence rescues the suppression of overall prostatic tumor growth by miR-7 restoration. A. KLF4 rescue improves cell proliferation by CCK8 test and cell number count. **B.** Rescue of KLF4 increases 2-D colony formation. **C.** KLF4 rescue recovers sphere formation in 3-D culture. Magnification: ×100; ×400, Bar: 50 μm. **D.** Rescue of KLF4 enhances tumorigenesis *in vivo*. **E.** KLF4 rescue promotes tumor growth in PC3-shKLF4 derived xenograft. Black arrow: tumor vessel. T: tumor. Magnification: ×100; ×400, Bar: 20 μm. Data are represented as mean ± SEM. *:$p < 0.05$; **:$p < 0.01$.

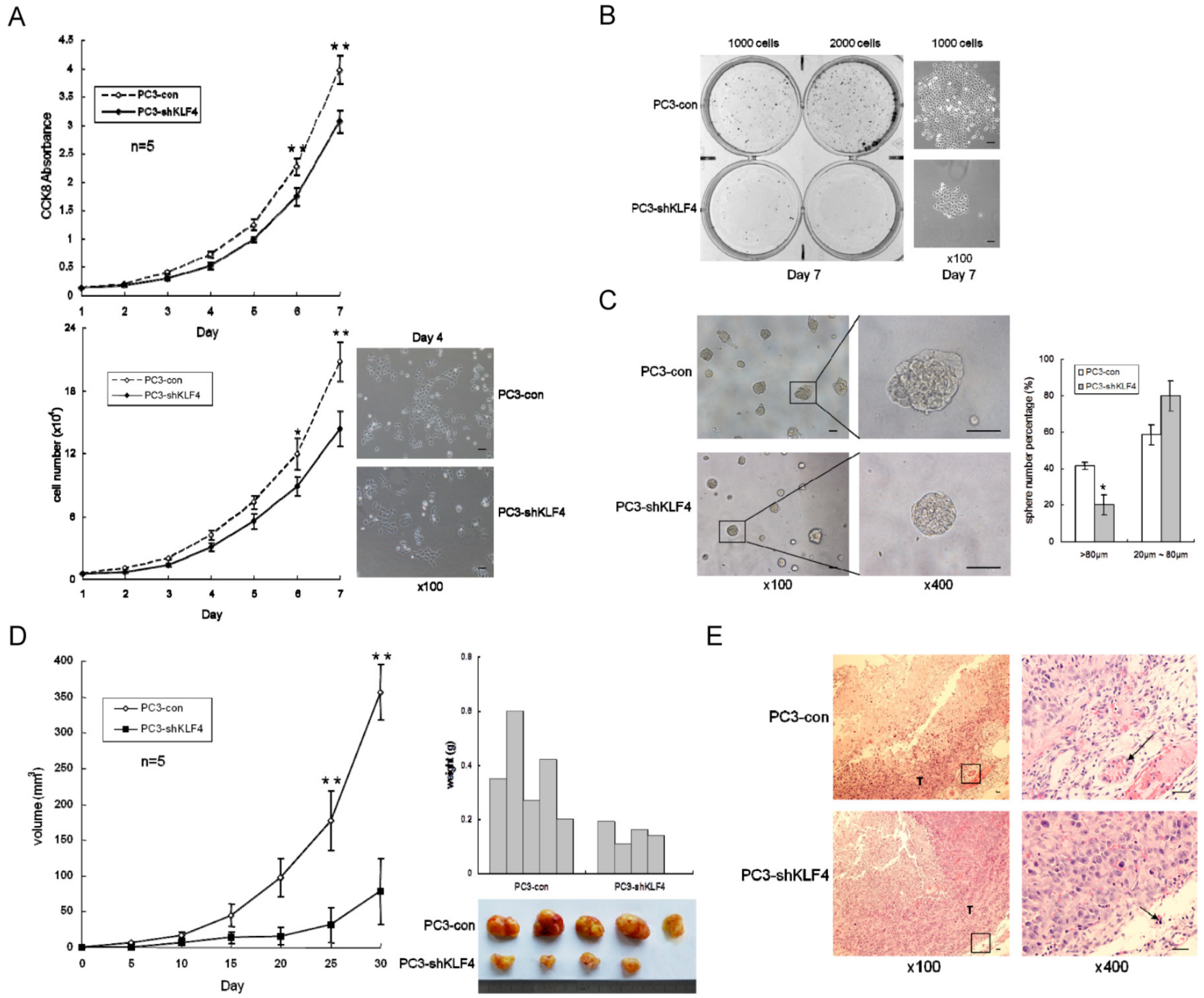

Supplementary Figure 7: Knock-down of KLF4 directly inhibits prostatic tumorigenesis. A. Knock-down of KLF4 directly inhibits cell proliferation by CCK8 test and cell number count. **B.** Knock-down of KLF4 directly inhibits 2-D colony formation. Magnification: ×100, Bar: 50 µm. **C.** Knock-down of KLF4 directly inhibits sphere formation in 3-D culture. Magnification: ×100; ×400, Bar: 50 µm. **D.** Knock-down of KLF4 directly decreases tumorigenesis *in vivo*. **E.** Knock-down of KLF4 directly inhibits tumor growth in PC3-shKLF4 derived xenograft. Black arrow: tumor vessel. T: tumor. Magnification: ×100; ×400, Bar: 20 µm. Data are represented as mean ± SEM. *:$p < 0.05$; **:$p < 0.01$.

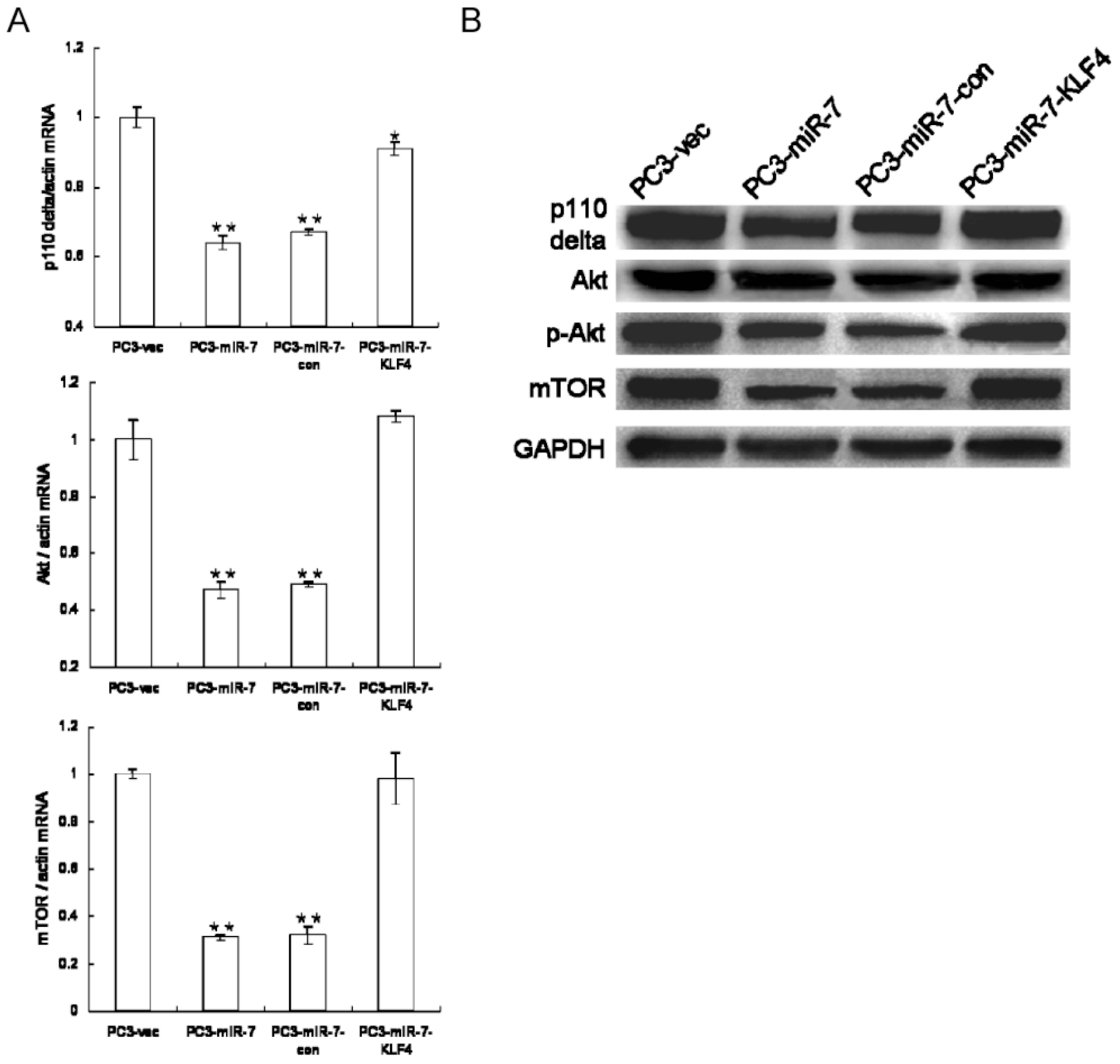

Supplementary Figure 8: KLF4 rescue augments PI3K/Akt signaling pathway. A. and **B.** Rescue of KLF4 up-regulates the expression of key components of the PI3K/Akt pathway, such as p110 delta, Akt and mTOR at both mRNA (A) and protein levels (B), which is suppressed by miR-7 restoration. Data are represented as mean ± SEM. *:$p < 0.05$; **:$p < 0.01$.

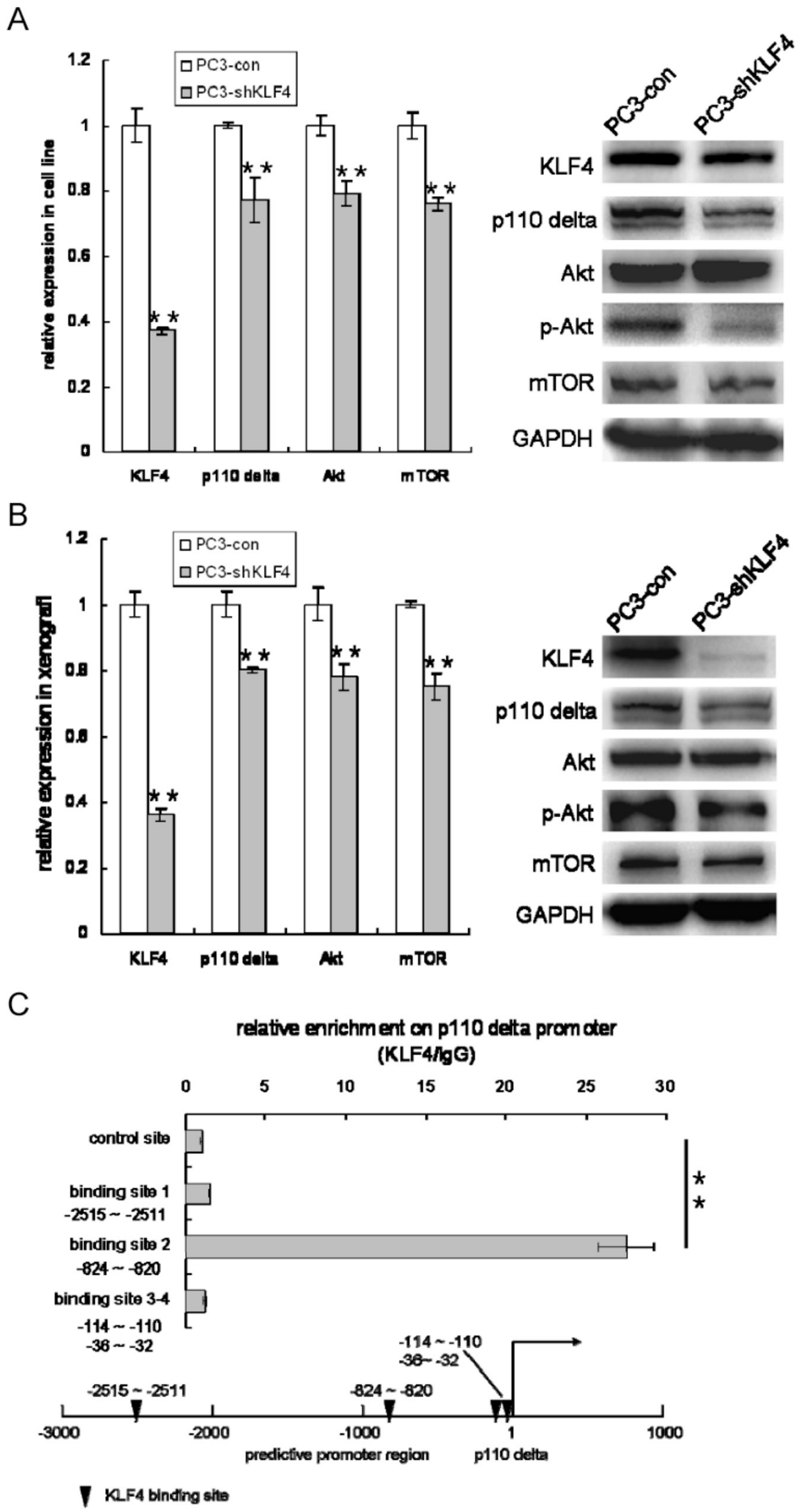

Supplementary Figure 9: KLF4 knock-down directly inhibits PI3K/Akt pathway and increases nuclear location of p21. A. and **B.** Knock-down of KLF4 directly inhibits expression of both KLF4 and PI3K/Akt pathway *in vitro* (A) and *in vivo* (B). **C.** Bioinformatic assay and ChIP sequencing demonstrates an interaction between KLF4 and promoter region of p110δ via four binding sites, which indicates that KLF4 regulates the transcription of p110δ, and in turn the expression of PI3K/Akt pathway. Data are represented as mean ± SEM. **:$p < 0.01$.

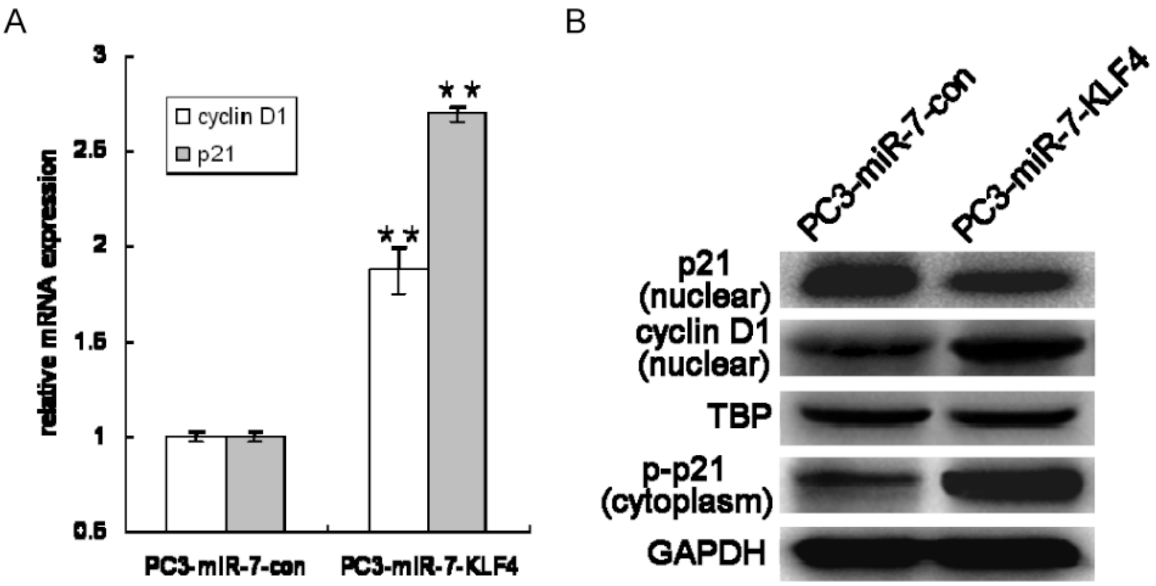

Supplementary Figure 10: Rescue of KLF4 increases nuclear localization of p21 and up-regulates cyclin D1 expression. A. The expression of p21 and cyclin D1 is up-regulated after KLF4 rescue. **B.** The phosphorylation of p21 in cytoplasm is increased and nuclear localization of p21 is decreased after KLF4 rescue. Data are represented as mean ± SEM. **:$p < 0.01$.

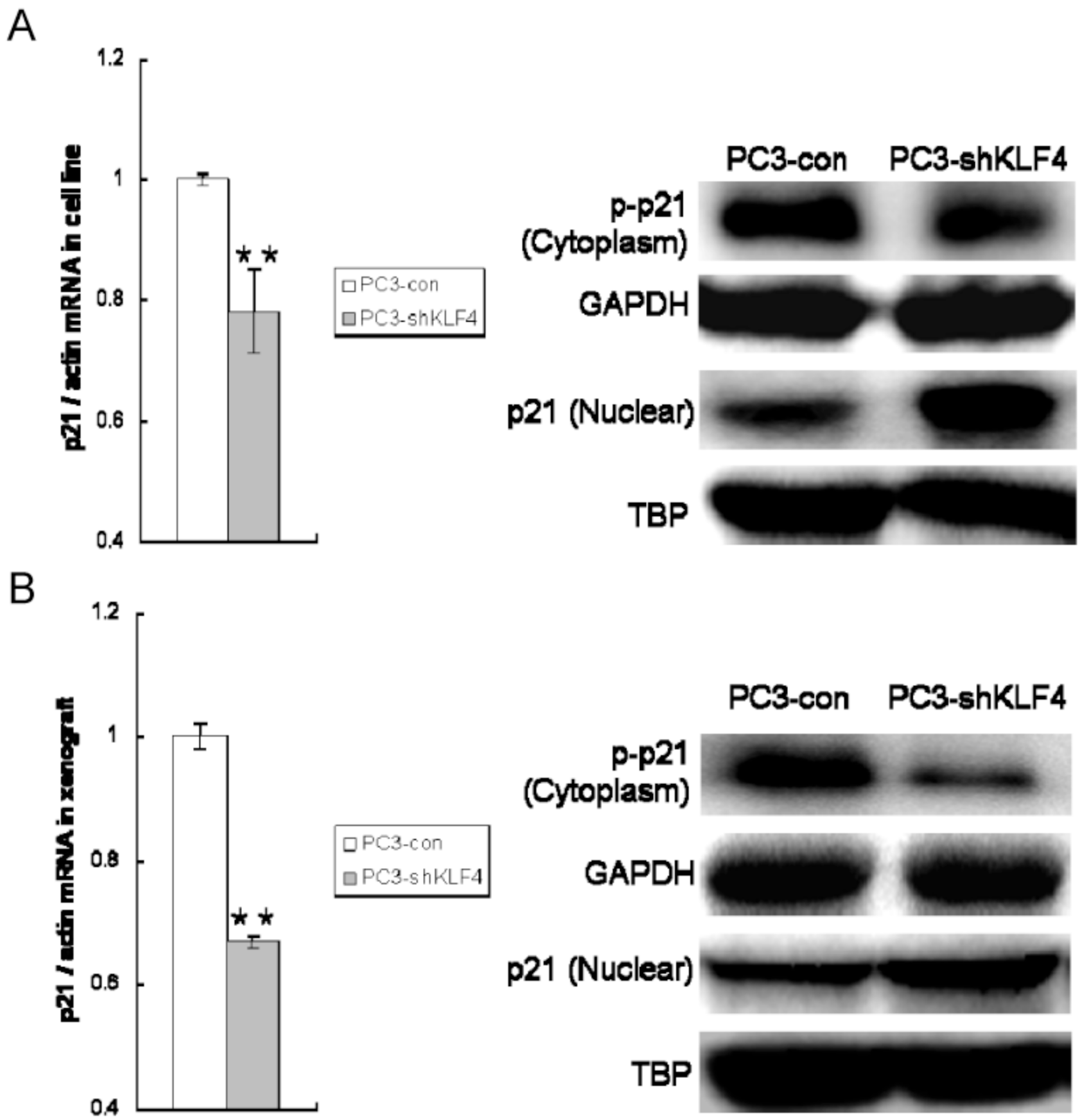

Supplementary Figure 11: KLF4 knock-down directly inhibits p21 expression but increases nuclear localization of p21. A. and **B.** Knock-down of KLF4 directly decreases the expression and phosphorylation of p21 and increases nuclear localization of p21 in the related cell line (A) *in vitro* and graft (B) *in vivo*. TBP: TATA-binding protein, internal control for nuclear protein. Data are represented as mean ± SEM. **:$p < 0.01$.

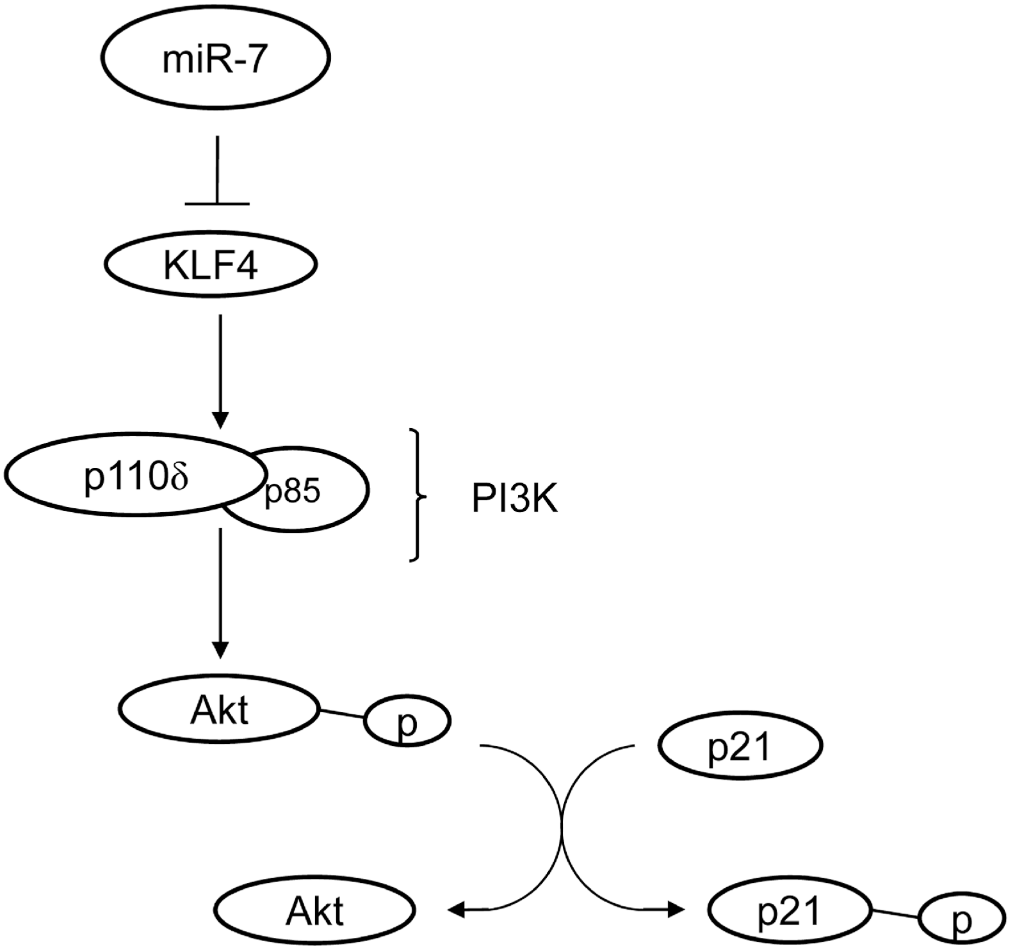

Supplementary Figure 12: MiR-7 inhibits stemness of PCSCs and prostate tumorigenesis via KLF4/PI3K/Akt/p21 pathway. MiR-7 inhibits KLF4 expression and its downstream PI3K/Akt/p21 pathway and increases nuclear localization of p21, which leads to inhibition of stemness of PCSCs and prostatic tumorigenesis.

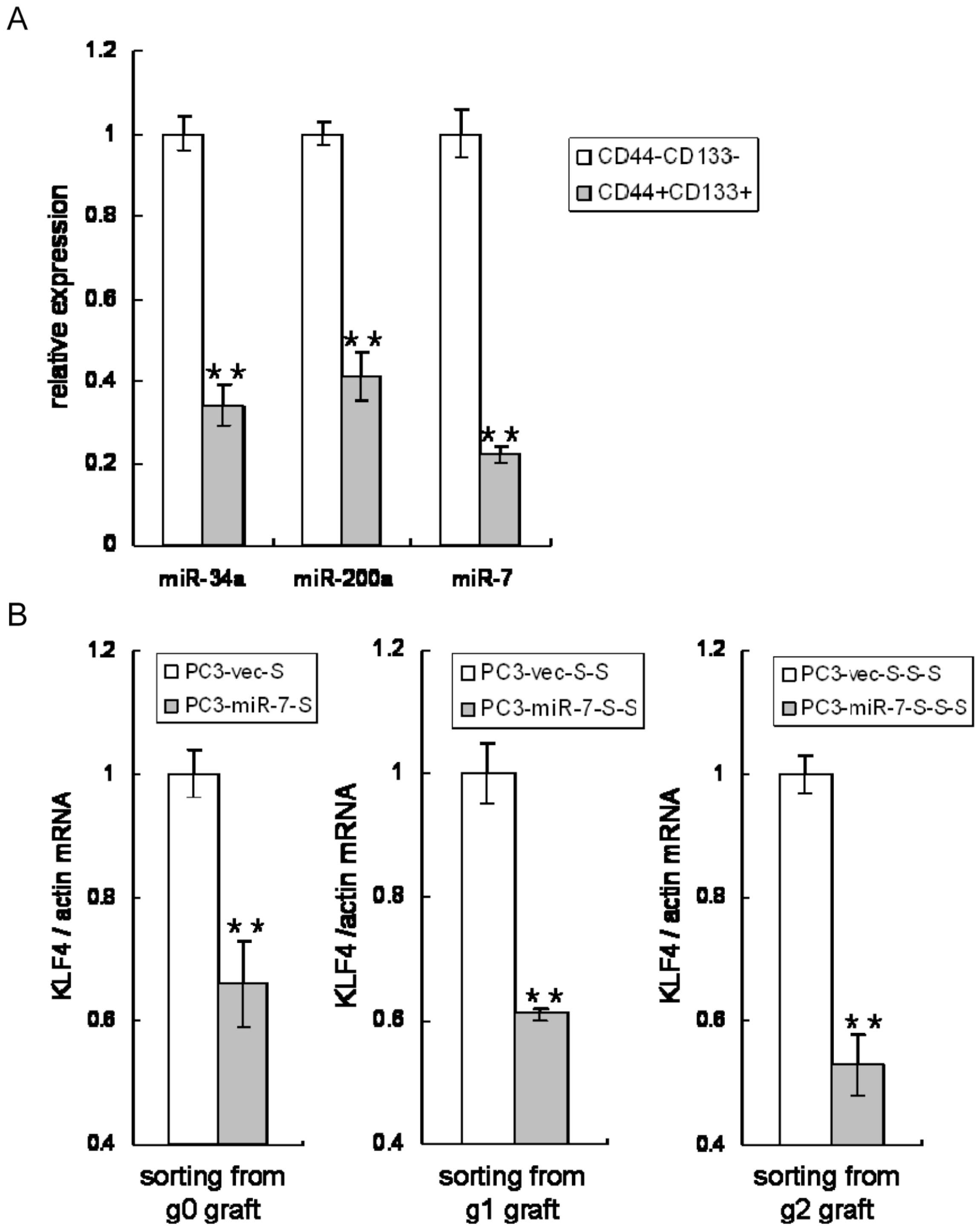

Supplementary Figure 13: MiR-7 functions as an inhibitor of PCSCs' stemness by continuously suppressing KLF4 expression for generations. A. Expression of miR-34a, miR-200a and miR-7 is significantly suppressed in CD44+CD133+ subpopulation. Among them the expression of miR-7 appears more suppressed than the other two. **B.** Expression of KLF4 is continuously down regulated in PCSCs sorting from g0, g1 and g2 grafts respectively, which indicates a stable inhibition of stemness by miR-7 restoration in PCSCs. Data are represented as mean ± SEM. **:$p < 0.01$.

Supplementary Table 1: Gleason Scores and associated clinical data from 20 patients in the study

	Gleason Score	TNM stage	PSA (ng/ml)	KLF4 (tumor/control)	miR-7 (tumor/control)
P1	7	T2cN0M0	13.62	1.35	3.02
P2	7	N/A	16.6	1.69	1.97
P3	6	T1cN0M0	4.16	1.61	0.5
P4	7	T2cN0M0	11.7	4.02	0.06
P5	6	T2bN0M0	16.02	2.05	0.24
P6	8	T3aN0M0	8.7	1.24	0.76
P7	7	N/A	11.3	1.21	5.86
P8	8	T2cN0M0	25.92	0.76	7.47
P9	7	T2cN0M0	7.49	2.42	0.17
P10	7	T2cN0M0	20.97	2.66	1.23
P11	6	T2bN0M0	20.85	1.43	0.74
P12	7	T2bN0M0	9.27	2.93	0.03
P13	7	T2cN0M0	8.78	4.06	0.05
P14	7	N/A	24	1.16	0.77
P15	7	T2cN0M0	45.21	3.92	0.06
P16	6	N/A	34	1.89	0.36
P17	6	N/A	7.8	0.85	6.83
P18	8	T3aN0M0	82.96	5.07	0.01
P19	6	N/A	6.23	2.1	1.5
P20	7	N/A	15.55	3.17	0.12

N/A: data not confirmed
